# Combinations of *Terminalia bellirica* (Gaertn.) Roxb. and *Terminalia chebula* Retz. Extracts with Selected Antibiotics Against Antibiotic-Resistant Bacteria: Bioactivity and Phytochemistry

**DOI:** 10.3390/antibiotics13100994

**Published:** 2024-10-19

**Authors:** Gagan Tiwana, Ian Edwin Cock, Matthew James Cheesman

**Affiliations:** 1 School of Pharmacy and Medical Sciences, Gold Coast Campus, Griffith University, Gold Coast 4222, Australia; g.tiwana@griffith.edu.au; 2 School of Environment and Science, Nathan Campus, Griffith University, Brisbane 4111, Australia; i.cock@griffith.edu.au

**Keywords:** MRSA, ESBL, plant extracts, natural therapies, combinational therapies, metabolomics

## Abstract

Antimicrobial resistance (AMR) has arisen due to antibiotic overuse and misuse. Antibiotic resistance renders standard treatments less effective, making it difficult to control some infections, thereby increasing morbidity and mortality. Medicinal plants are attracting increased interest as antibiotics lose efficacy. This study evaluates the antibacterial activity of solvent extracts prepared using *Terminalia bellirica* and *Terminalia chebula* fruit against six bacterial pathogens using disc diffusion and broth microdilution assays. The aqueous and methanol extracts of *T. bellirica* and *T. chebula* showed substantial zones of inhibition (ZOIs) against *Staphylococcus aureus* and methicillin-resistant *S. aureus* (MRSA). The activity against those bacteria was strong, with minimum inhibitory concentrations (MIC) ranging from 94 µg/mL to 392 µg/mL. Additionally, the *T. bellirica* methanolic extract showed noteworthy antibacterial activity against *Escherichia coli* and an extended spectrum β-lactamase (ESBL) *E. coli* strain (MIC values of 755 µg/mL for both). The aqueous *T. bellirica* and *T. chebula* extracts also inhibited *Klebsiella pneumoniae* growth (MIC values of 784 µg/mL and 556 µg/mL, respectively). The corresponding methanolic extracts also inhibited ESBL *K. pneumoniae* growth (MIC values of 755 µg/mL and 1509 µg/mL, respectively). Eighteen additive interactions were observed when extracts were combined with reference antibiotics. Strong antagonism occurred when any of the extracts were mixed with polymyxin B. Liquid chromatography-mass spectroscopy (LC-MS) analysis of the extracts revealed several interesting flavonoids and tannins, including 6-galloylglucose, 1,2,6-trigalloyl-β-D-glucopyranose, 6-O-[(2E)-3-phenyl-2-propenoyl]-1-O-(3,4,5-trihydroxybenzoyl)-β-D-glucopyranose, propyl gallate, methyl gallate, sanguiin H4, hamamelitannin, pyrogallol, gallic acid, ellagic acid, chebulic acid, and chebuloside II. All extracts were nontoxic in brine shrimp assays. This lack of toxicity, combined with their antibacterial activities, suggests that these plant species may be promising sources of antibacterial compound(s) that warrant further study.

## 1. Introduction

Antimicrobial resistance (AMR) is one of the most concerning public health issues of the 21st century. The development of AMR mechanisms in bacterial pathogens leads to lower efficacies for standard antimicrobial therapies, which in turn promotes the transmission of these infections. The World Health Organization (WHO) has highlighted antimicrobial resistance (AMR) as a major danger to global health, food security, and future development, with far-reaching effects on human health and economic stability [1]. 

Antibiotic-resistant bacterial strains have rapidly arisen due to antibiotic misuse and overuse in humans and animals, as well as in agriculture. Antibiotic misuse includes excessive antibiotic prescriptions, poor treatment compliance, and the widespread use of antibiotics in livestock for growth promotion and disease prevention. This results in longer illness durations, higher mortality rates, and elevated healthcare costs [1]. AMR mortality and morbidity are concerning. AMR caused 1.27 million deaths worldwide in 2019, and current trends indicate that this number will climb dramatically [1]. Infections caused by antibiotic-resistant bacteria, such as methicillin-resistant *Staphylococcus aureus* (MRSA) and multidrug-resistant *Escherichia coli*, are particularly concerning due to their high resistance rates and severe clinical outcomes [2]. The costs associated with AMR are multifaceted, encompassing direct medical costs such as longer hospital stays and more intensive care, as well as indirect costs such as lost productivity and long-term disability. According to the World Bank, by 2050, AMR is likely to cause a loss of global economic output amounting to USD 1 trillion annually, with healthcare costs also projected to increase significantly [3]. 

The 2022 Global Antimicrobial Resistance and Use Surveillance System (GLASS) report underscores the significant concern regarding the alarming resistance rates of third-generation cephalosporin-resistant *E. coli* and MRSA in 76 countries [1,4]. That report also found a significant rise in *E. coli* infections resistant to ampicillin, co-trimoxazole, and fluoroquinolones. The common intestinal bacterium *Klebsiella pneumoniae* has also shown increased resistance to penicillins, cephalosporins and fluoroquinolones. These elevated levels of antibiotic resistance may lead to an increase in the use of last-resort medications, such as carbapenems [1,4]. The rise of antibiotic-resistant bacteria has outpaced drug development. The high expenditures and limited financial returns of antibiotic research and development have caused a sharp fall in the number of novel antibiotics approved for clinical use [5]. According to the WHO, there is a concerning lack of new antibacterials in the pipeline due to the lengthy research and development processes, and the high probability of failure during clinical testing [6]. Thirteen new antibiotics have received therapeutic authorization since July 2017. However, only two of these antibiotics constitute a new chemical class and are considered innovative [6]. This highlights the significant scientific and technical difficulties in finding new antibacterials that are both effective against bacteria and safe for human use. 

Exploring the potential of medicinal plants in the development of antimicrobial drugs presents numerous benefits compared to conventional antibiotics. Traditional medicinal plants offer an accessible and cost-effective alternative, requiring fewer resources and less time for production [7]. They often interact with the body in a safe manner, causing minimal side effects, and often have complementary effects that enhance overall well-being. However, medicinal plants can also cause toxicity if misused. Therefore, careful dosing, accurate identification, and monitoring are essential to avoid adverse effects. Phytochemicals in medicinal plants may work together to boost antibacterial efficacy, potentiating the treatment’s efficacy [7,8]. Medicinal plants possess a variety of mechanisms of action, which holds great promise in combating antibiotic-resistant bacteria [8]. The combination of plant extracts/compounds with conventional antibiotics is a strategy that can be utilized in the development of new antimicrobial therapies [9]. Phytochemicals may enhance antibiotic efficacies through synergistic and additive interactions, potentially overcoming resistance issues. This approach offers a promising solution for developing effective treatments against antibiotic-resistant bacterial strains. For example, propolis ethanolic extract potentiates the efficacy of ampicillin and vancomycin against the methicillin-resistant *S. aureus* (MRSA) [10]. 

The current study focuses on the antibacterial activity, phytochemistry, and toxicity of fruit extracts (aqueous, methanol, and ethyl acetate) derived from *Terminalia bellirica* (Gaertn.) Roxb. and *Terminalia chebula* Retz. Antibiotic-sensitive and antibiotic-resistant bacterial pairs of *S. aureus*, *E. coli*, and *K. pneumoniae* were examined for their growth in the presence of the extracts, as well as a panel of reference antibiotics. Our previous work revealed enhancements of *E. coli* antibacterial activity when *T. bellirica* and *T. chebula* fruit extracts were combined [11]. However, antibiotic-resistant bacterial species were not tested in that study, nor were combinational interactions between pure antibiotics and the plant extracts examined. Thakur et al. (2024) recently identified good antibacterial activities of the *T. bellirica* aqueous fruit extract against *S. aureus, E. coli*, and *E. coli* 385 (an antibiotic-resistant strain), with minimum inhibitory concentrations (MICs) of 312 µg/mL, 1250 µg/mL and 625 µg/mL against these species, respectively [12]. Phytochemical analysis was conducted by liquid chromatography–mass spectrometry (LC-MS), which identified a single polyphenol compound, bergenin [12]. Interestingly, flavonoids, tannins and terpenoids were not identified in the *T. bellirica* fruit extracts in that study. Furthermore, combinational interactions between reference antibiotics and the extracts were not explored. In addition, prior studies have demonstrated that fruit extracts of *T. bellirica* exhibit antibacterial activity against *S. aureus*, *E. coli*, and *K. pneumoniae* [13,14,15]. However, those investigations did not include combinational studies of extracts with antibiotics and testing of antibiotic-resistant bacterial strains. Other investigations reported inconsistent results for *T. chebula* fruit extracts (aqueous, ethanol, and dimethylformamide) against *S. aureus* and *K. pneumoniae*, with ZOIs of 15–18 mm on agar, using 500 µg extract per disc [16]. Furthermore, low-to-inactive antibacterial activities of *T. chebula fruit* extracts were observed against antibiotic-sensitive and antibiotic-resistant strains of *S. aureus* and *E. coli* [17,18].

In order to address the discrepancies in the previous studies regarding the activities of *T. bellirica* and *T. chebula*, and the absence of thorough phytochemical analyses of the extracts, we investigated the antibacterial properties of aqueous, methanolic, and ethyl acetate fruit extracts obtained from *T. bellirica* and *T. chebula*. Our study focused on selected bacterial pathogens, but included resistant strains of *S. aureus*, *E. coli*, and *K. pneumoniae*. We conducted disc diffusion and liquid microdilution assays to assess their effectiveness. Additionally, LC-MS was utilized to examine the phytochemical composition of *T. bellirica* and *T. chebula* and to identify specific flavonoid, phenolic acid, terpenoid, and tannin molecules. In addition, our study investigated the interactions between active plant extracts and a panel of reference antibiotics when tested as extract–antibiotic combinations in MIC assays. Finally, the toxicities of the extracts were assessed using *Artemia franciscana* Kellogg nauplii lethality assays to provide initial insights into the safety of the extracts for possible clinical use.

## 2. Results

### 2.1. Antimicrobial Susceptibility Assays

Individual 1 g masses of *T. bellirica* and *T. chebula* fruit powder was extracted separately using sterile deionized water, methanol, or ethyl acetate. The extracts were subsequently dried and resuspended in 10 mL of 1% DMSO. The extract concentrations for the two plants were generally similar, where the highest yields were obtained using methanol as a solvent, whilst ethyl acetate produced very low yields for each plant. Specifically, *T. bellirica* extract yields for water, methanol and ethyl acetate solvent extractants were 50.2, 48.3, and 3.6 mg/mL, respectively, whilst for *T. chebula* they were 35.6, 48.3, and 4.9 mg/mL. 

**Figure 1:**
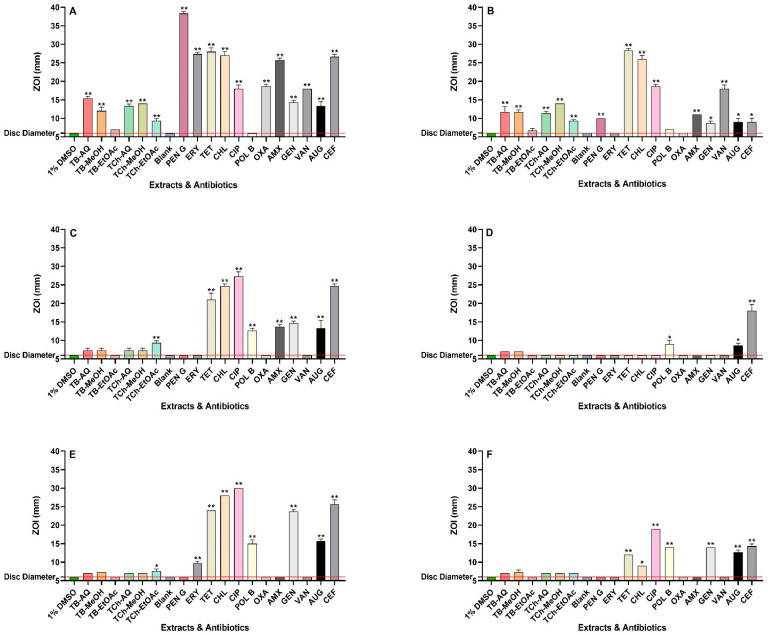
Antimicrobial activities of *T. bellirica* and *T. chebula* fruit extracts in agar disc diffusion assays against (**A**) *S. aureus*, (**B**) MRSA, (**C**) *E. coli*, (**D**) ESBL *E. coli*, (**E**) *K. pneumoniae*, and (**F**) ESBL *K. pneumoniae*. TB-AQ = *T. bellirica* aqueous; TB-MeOH = *T. bellirica* methanol; and TB-EtOAc = *T. bellirica* ethyl acetate. TCh-AQ = *T. chebula* aqueous; TCh-MeOH = *T. chebula* methanol; and TCh-EtOAc = *T. chebula* ethyl acetate. Negative controls = 1% DMSO and Blank = sterile water. Reference antibiotics: PEN G = penicillin G (10 IU), ERY = erythromycin (10 µg), TET = tetracycline (30 µg), CHL = chloramphenicol (30 µg), CIP = ciprofloxacin (1 µg), POL B = polymyxin B (300IU), OXA = oxacillin (1 µg), AMX = amoxycillin (10 µL of 0.01 mg/mL stock solution), GEN = gentamicin (10 µg), VAN = vancomycin (30 µg), AUG = Augmentin^®^ (15 µg), and CEF = cefoxitin (30 µg). Horizontal red lines on the *y*-axis at 6 mm indicates the disc diameter used in the assay. Mean values (±SEM) are reported from three independent studies. *p*-values < 0.01 are represented with a single asterisk symbol (*), while *p*-values < 0.001 are represented with a double asterisk symbol (**).

The antibacterial efficacies of all extracts and reference antibiotics were evaluated using agar disc diffusion assays and broth microdilution assays. The aqueous and methanolic extracts of both plants exhibited activities varying from low to high against the six bacterial pathogens tested in the disc diffusion and broth dilution assays (Figure 1 and Table 1, respectively). The broth microdilution assays were used to quantify MIC values of the extracts and reference antibiotics (Table 1). The differences between the antibiotic-sensitive and antibiotic-resistant strains of the pathogens can be readily observed by the generally higher MIC values for many of the antibiotics against the resistant bacteria compared to their sensitive counterparts, as well as the lower ZOIs resulting from agar disc diffusion assays. This demonstrates that the antibiotic-resistant strains are much less sensitive to many of the antibiotics tested in this study.

When aqueous and methanol extracts of *T. bellirica* were evaluated against *S. aureus* and MRSA, they exhibited MIC values ranging from 94 and 392 µg/mL, respectively. The *T. bellirica* ethyl acetate extract also inhibited *S. aureus* and MRSA growth, albeit with a higher MIC value of 900 µg/mL. In contrast, all *T. chebula* extracts exhibited good antibacterial activity against *S. aureus* and MRSA, with MIC values ranging from 139 µg/mL to 306 µg/mL. Concordant findings were obtained for both plant extracts against *S. aureus* and MRSA in agar disc diffusion assays, where only small but detectable ZOIs were observed for the *T. bellirica* ethyl acetate extracts against the two *Staphylococcus* species, although much larger ZOIs of 9–15 mm were produced by all other extracts against *S. aureus* and MRSA (Figure 1).

Similarly, the extracts were also screened against *E. coli* and *K. pneumoniae*, as well as their antibiotic-resistant ESBL counterparts. The agar disc diffusion experiments showed limited growth inhibition for most of the extracts when tested against *E. coli*, ESBL *E. coli*, *K. pneumoniae*, and ESBL *K. pneumoniae*. However, these results cannot be considered as zones of inhibition (ZOIs) because complete inhibition surrounding the disc is necessary. Interestingly, the *T. chebula* ethyl acetate extract produced a small but distinct ZOI against *E. coli* and *K. pneumoniae* on agar (7–9 mm) in the disc diffusion assay (Figure 1). Furthermore, relatively high MIC values were determined for the *T. bellirica* aqueous extracts and the *T. chebula* aqueous and methanol extracts against *E. coli* and ESBL *E. coli* (MIC = 1509 µg/mL to 3138 µg/mL). Notably, an MIC value of 755 µg/mL was obtained for the *T. bellirica* methanolic extract against these two species. The aqueous *T. bellirica* and *T. chebula* extracts also exhibited noteworthy antibacterial activity against *K. pneumoniae*, with MIC values of 784 µg/mL and 556 µg/mL, respectively. Moderate activities towards this bacterium were found when the *T. bellirica* and *T. chebula* methanolic extracts were tested (MIC = 1509 µg/mL), as well as the ethyl acetate *T. chebula* extract (MIC = 1225 µg/mL). Additionally, the ESBL *K. pneumoniae* was inhibited in broth assays by the methanolic *T. bellirica* and *T. chebula* extracts (MIC values of 755 µg/mL and 1509 µg/mL, respectively). However, the aqueous extracts yielded low activities (relatively high MIC values) against ESBL *K. pneumoniae* (MIC values of 3138 µg/mL and 2225 µg/mL, respectively). 

### 2.2. Combination Assays: Sum of Fractional Inhibitory Concentration (ƩFIC) Determinations

Combinations of the *T. bellirica* and *T. chebula* extracts with conventional antibiotics were tested to detect any possible interactions between the two components against the antibiotic-sensitive and antibiotic-resistant bacterial strains (Table 2). For these assays to be conducted, both the extract and the antibiotic must demonstrate efficacy against the bacterial species under examination, as MIC values for both components are required to determine ƩFIC values. No synergistic interactions were observed for any of the combinations. Eighteen combinations caused additive effects, and eighty combinations were indifferent. Additionally, nineteen combinations had antagonistic effects. 

### 2.3. Compound Identification by LC-MS Metabolomics Fingerprinting

LC-MS fingerprinting analysis was employed to investigate the metabolomic fingerprints of all extracts, with particular focus on the flavonoid, tannin, and terpenoid compounds. Most of the extract compounds eluted in the gradient stage of the chromatogram from 30% to 90% acetonitrile (Supplementary Materials Figures S1 and S2), suggesting that most of the extract components were of relatively high polarity [19]. Organic acids and amines are more likely to elute in polar environments and therefore do so early in the chromatogram. Conversely, lipophilic substances and hydrocarbons, which exhibit a stronger interaction with the non-polar stationary phase, elute later as the gradient persists. Only compounds that were completely matched in any of the databases (and were not present in the blank controls) were chosen to create a potential inventory of all the identified compounds (Supplementary Materials: Tables S1 and S2). In this study, we have focused on the flavonoid, tannin, terpenoid and phenolic acid compounds (Table 3). 

### 2.4. Toxicity Quantification

The plant extracts were evaluated for toxicity in triplicate assays conducted on 48-well plates using *Artemia franciscana* Kellogg nauplii lethality assays (ALA). The toxicity level was determined, and plant extracts were classified as toxic if their LC_50_ values were less than 1000 µg/mL following 24 h exposure [11]. More than half of the nauplii evaluated in this study remained alive for all extracts after their 24 h duration of incubation. Consequently, all plant extracts tested were classified as nontoxic. Indeed, the outcomes of all extracts were comparable to those of the negative control (artificial seawater).

## 3. Discussion

Our study tested *T. bellirica* and *T. chebula* fruit extracts against a group of bacterial pathogens that are significant for human health, including strains that are resistant to antibiotics. Significantly, the aqueous and methanolic extracts effectively suppressed the growth of the six bacterial pathogens examined in this study, underscoring their potential as targets for plant-based antibiotics development. The methanolic extracts exhibited the highest antibacterial efficacy in both the disc diffusion and liquid microdilution experiments against all pathogens. The ethyl acetate *T. bellirica* and *T. chebula* extracts also exhibited good antibacterial activities against *S. aureus* and MRSA in the disc diffusion and broth microdilution assays. In addition, the *T. chebula* ethyl acetate extract showed moderate antibacterial efficacy against *K. pneumoniae*. The differences in net yields and the populations of phytochemicals extracted by different solvents may account for these variances in potency. Methanol and water have a higher polarity compared to ethyl acetate, which results in the extraction of a larger quantity of phytochemicals with high-to-mid polarity. In contrast, ethyl acetate extracts a smaller number of compounds with mid-to-lower polarity [20]. The differences in phytochemical contents among the extracts may potentially contribute to the varied antibacterial growth inhibition effects observed in the disc diffusion and liquid dilution investigations. Phytochemicals that have lower polarity or are larger in size diffuse less rapidly through solid agar, resulting in a decrease in their apparent antibacterial effectiveness in disc diffusion assays [21]. The solubility of these phytochemicals in broth is also influenced by their polarity [22], which in turn impacts their ability to dissolve and may lead to inaccurate MIC values. Prior research has demonstrated that the depth of agar in petri dishes, as well as the regularity of the agar, may impact the extent of zones of inhibition (ZOIs) in agar diffusion studies [23]. Whilst we followed manufacturers’ instructions to create agar of uniform consistency and poured it at a consistent depth of 4 mm, repeating the disc diffusion experiments at varying agar depths may be useful to confirm the ZOIs of all the extracts and antibiotics. In this investigation, we noticed that the borders of the zone of inhibition (ZOI) were clearly visible for *S. aureus* and MRSA, although they were less clear (regarded as no ZOI) for the other bacterial pathogens studied. It is important to note that in future research, the use of methylene blue or crystal violet to stain the plates may enhance the visibility of ZOIs if there is low clarity [24]. 

Notably, the MRSA strain tested herein showed resistance to many commonly used antibiotics, such as β-lactams (penicillin G, oxacillin, amoxicillin), and macrolides (erythromycin). Nevertheless, the emergence of extended spectrum β-lactamase enzymes has made these medications less effective against some bacterial strains. Likewise, macrolide antibiotics such as erythromycin are commonly employed because they have the ability to impede protein synthesis in bacteria [25]. The existence of MRSA resistance to macrolides complicates the availability of treatment options, highlighting the need for new therapeutic drugs and procedures. Hence, it is imperative to identify innovative compounds that can elude or surmount these resistance mechanisms. The antibacterial activities observed with *T. bellirica* and *T. chebula* extracts against *S. aureus* and MRSA produced similar MIC values ranging from 94 µg/mL to 900 µg/mL. These findings indicate that the resistance mechanisms observed in the MRSA strain have little influence on the active compounds of the extracts. Thus, the extract compounds either function through different mechanisms, or they may inhibit the bacterial antibiotic-resistance pathways. 

The *mecA* gene plays a crucial role in the resistance of MRSA [26]. This gene codes for a new penicillin-binding protein (PBP2a) that has a reduced ability to bind to β-lactam antibiotics. This protein provides the bacteria with resistance against many β-lactam antibiotics by enabling them to produce cell walls, even in the presence of these medications. Hence, the mechanisms by which the phytochemicals in the extract function may vary from those of β-lactam antibiotics, even in bacteria that are resistant to β-lactam antibiotics. Alternatively, these extracts may contain phytochemicals that disrupt the bacterial strains’ defense mechanisms against these medicines, thereby allowing them to function at higher potency [26]. This outcome is promising because, when compared to the susceptible strain, the MRSA strain in our study exhibited significantly diminished susceptibilities/increased resistance to a range of antibiotics from the β-lactams, macrolides and fluoroquinolones classes. 

Aqueous and methanol *T. bellirica* and *T. chebula* extracts exhibited antibacterial activity against *E. coli* and its antibiotic-resistant counterpart, ESBL *E. coli*. The methanolic *T. bellirica* extract showed noteworthy antibacterial activity against both bacterial pathogens, with identical MIC values. Similarly, the methanolic *T. bellirica* and *T. chebula* extracts also inhibited the growth of *K. pneumoniae* and ESBL *K. pneumoniae*, with similar MIC values. These results suggest that the methanolic extracts of both plants may contain compounds that have a broad efficacy against strains of *E. coli* and *K. pneumoniae*, including those that produce ESBL enzymes. Their efficacy might be linked to a distinct mechanism of action that contrasts those of β-lactam antibiotics. For example, the plant extracts may affect how bacteria form their cell walls, how well their membrane’s function, or they may affect other essential processes independent of the β-lactam antibiotic mechanism [27]. In addition, it is possible that the methanolic extracts of both plants are exerting antibacterial effects through mechanisms that do not directly interfere with or inhibit the ESBL enzymes. Additional research is necessary to ascertain whether the plant extracts specifically hinder the resistance mechanisms of ESBL bacterial pathogens, or if they function via independent antibiotic pathways. This may require assessment of the effects of the extract/isolated components on β-lactamase inhibition, or by investigating the impact of the extract on the production of resistance genes in ESBL strains. 

We also examined the use of *T. bellirica* and *T. chebula* extracts in combinations with conventional antibiotics. This method holds great promise for developing novel antibiotic chemotherapies, as many bacteria have developed resistance to conventional antibiotics, and plant compounds may provide ways of inhibiting/blocking these resistance mechanisms [4]. Our goal was to enhance the efficacy of antibiotics and possibly negate the bacterial resistance mechanisms by mixing them with extracts from plants. Augmentin^®^, a combination of amoxicillin and clavulanic acid, is a well-known example of how combining medications can improve treatment outcomes [28] since clavulanic acid inhibits β-lactamase enzymes present in resistant bacteria. This enables amoxicillin to specifically target and eliminate the bacteria with greater efficiency, even in β-lactam-resistant bacterial strains. Clavulanic acid acts as a β-lactamase inhibitor by binding permanently to the active site of the enzyme, therefore inhibiting the breakdown of the antibiotic. 

Our investigation found that penicillin G, amoxicillin, in combination with the plant extracts, have additive effects against *S. aureus*, which may be due to the presence of phytochemicals that possess anti-β-lactamase activities [29]. These phytochemicals can hinder the activity of β-lactamase enzymes, thereby contributing to antibiotic resistance by breaking down the β-lactam ring found in β-lactam antibiotics, such as penicillin and amoxicillin [30]. In addition, phytochemicals found in the plant extracts may interact with β-lactamase enzymes in a way that is comparable to clavulanic acid, although this requires confirmation. Regardless of the mechanism, this interaction helps protect the antibiotics from being broken down by bacterial enzymes and improves their ability to fight against bacterial pathogens. Plant extracts may possess the capacity to function as an alternative treatment method and could be a safe and less detrimental option for addressing antibiotic resistance [9]. In comparison to synthetic antibiotics, natural compounds found in plants frequently exhibit fewer adverse effects and a lower likelihood of generating resistance [8]. Moreover, the likelihood of further antibiotic-resistance development may be reduced by the variety of phytochemicals present in plant extracts, which may act on multiple bacterial pathways.

The ethyl acetate *T. chebula* extract exhibited additive effects against *S. aureus* in combination with erythromycin. It is possible that the extract and erythromycin may target distinct bacterial processes. Erythromycin inhibits protein synthesis by binding to the 50S ribosomal subunit [31], whereas the plant extract may impact other bacterial targets, including cell wall synthesis, membrane integrity, or metabolic processes [30]. Further studies are required to determine which of these mechanisms are affected by the extracts. Notably, this complementary targeting has the potential to improve the overall antibacterial effect. Furthermore, the plant extract may also influence other mechanisms associated with bacterial resistance, such as the disruption of efflux pumps or the modification of cell wall structures [30], which could render bacteria more susceptible to erythromycin. The extract may also disrupt bacterial protective barriers, which could facilitate the improved penetration of erythromycin into the cell. Additionally, the plant extract may contain a variety of bioactive compounds [8] that work in conjunction to enhance the efficacy of erythromycin. Alternatively, the extract may exhibit broad-spectrum antimicrobial activity that complements the specific effects of erythromycin. Similarly, the methanolic and ethyl acetate extracts of *T. chebula* exhibited additional antibacterial effects against *K. pneumoniae* when combined with chloramphenicol. This antibiotic binds to the 23S rRNA component of the 50S ribosomal subunit of the bacterial ribosome [32]. This binding suppresses peptidyl transferase activity, a crucial process for the creation of peptide bonds during protein synthesis. Chloramphenicol inhibits bacterial protein production by blocking the formation of peptide bonds. 

Our research determined that when tetracycline is combined with *T. chebula* extracts, the combination exhibited additive interactions against *S. aureus*, MRSA, *E. coli* and *K. pneumoniae*, suggesting enhanced antibacterial efficacy. However, the aqueous *T. bellirica* extract only showed an additive interaction against MRSA. Tetracycline resistance is mostly commonly attributed to tetracycline-specific efflux pumps [33]. Hence, the observed additive effect indicates that the *T. bellirica* and *T. chebula* extracts might have hindered the functioning of these efflux pumps. By inhibiting efflux pumps, tetracycline is allowed to remain inside cells for a longer period, which increases its effectiveness. Whilst ribosomal changes may also contribute to tetracycline resistance, this pathway is substantially less common [33]. Our research findings suggest that a broad range of phytochemicals found in the extracts of *T. bellirica* and *T. chebula* may have antibacterial action against several different bacteria. Similarly, plant extracts prepared from *Phyllanthus niruri* L., *Berberis vulgaris* L., and *Piper nigrum* L. exhibit additive interactions in combination with tetracycline, and can inhibit tetracycline efflux pumps [34,35].

Interestingly, the *T. bellirica* and *T. chebula* ethyl acetate extracts exhibited additive interactions in combination with ciprofloxacin against MRSA. This indicates that ethyl acetate extracts contain phytochemicals that may target different bacterial processes such as cell membrane disruption, inhibition of cell wall synthesis, or interference with metabolic pathways [30]. The plant extract’s unique mechanisms may enhance the effectiveness of ciprofloxacin’s DNA-targeting action, resulting in a more comprehensive treatment [8]. The plant extract may also enhance the cellular absorption of ciprofloxacin (thereby increasing its intracellular concentration) or it may block/inhibit MRSA’s antibiotic-resistance mechanisms [35]. 

Notably, polymyxin B and *T. bellirica*, and *T. chebula* extracts combinations exhibited substantial antagonistic interactions. The antagonistic interaction between polymyxin B and the plant extracts may be affected by variations in pH levels in the broth. Polymyxin B, which breaks bacterial cell membranes by binding to lipopolysaccharides, is pH sensitive, with substantially reduced potency under acidic or alkaline circumstances [36]. The inclusion of plant extracts may cause a change in the pH of the broth, which could affect the efficacy of both polymyxin B and the plant chemicals, potentially resulting in a decrease in overall antibacterial activity, although this remains to be confirmed in future studies. Changes in pH may also impact bacterial physiology, thereby reducing the vulnerability of bacteria to the combined actions of the two substances. Polymyxin B and the components of plant extracts may also have chemical interactions that are regulated by pH [36], which may further contribute to the antagonistic effects of the combination. Moreover, the antagonistic interactions between the extracts and polymyxin B can be ascribed to the binding of bioactive phytochemicals with polymyxin B, which impedes its absorption and efficacy in targeting bacterial cells [37]. Hence, understanding these dynamics is critical for optimizing combination medicines and assuring antimicrobial efficacy. Future studies are planned to examine these effects.

**Figure 2:**
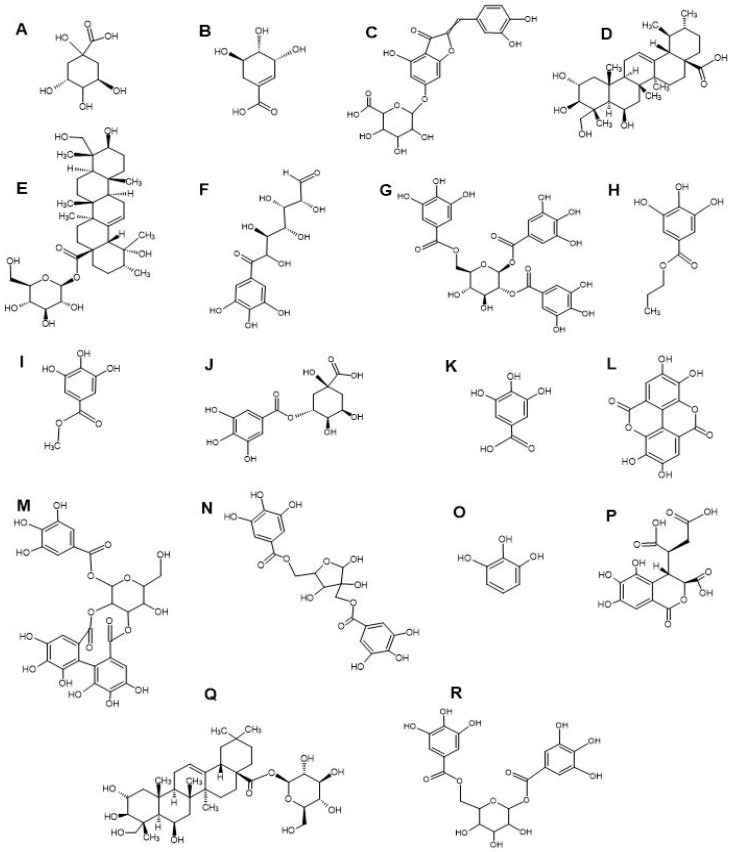
Structures of noteworthy compounds identified in the fruit extracts of *T. bellirica* and *T. chebula*. Quinic acid (**A**), shikimic acid (**B**), aureusidin 6-glucuronide (**C**), madecassic acid (**D**), pedunculoside (**E**), 6-galloylglucose (**F**), 1,2,6-trigalloyl-β-D-glucopyranose (**G**), propyl gallate (**H**), methyl gallate (**I**), theogallin (**J**), gallic acid (**K**), ellagic acid (**L**), sanguiin H4 (**M**), hamamelitannin (**N**), pyrogallol (**O**), chebulic acid (**P**), chebuloside II (**Q**), and 1,6-bis-O-(3,4,5-trihydroxybenzoyl) hexopyranose (**R**).

LC-MS metabolomics analysis of the *T. bellirica* and *T. chebula* fruit extracts highlighted the presence of flavonoids, tannins, terpenoids and phenolic acid compounds (Table 3). Complete, comprehensive lists of phytochemicals present in the individual plant extracts are available as the Supplementary Files (Supplementary Materials: Tables S1 and S2). Notable phytochemicals identified in both plant extracts include quinic acid (Figure 2A), shikimic acid (Figure 2B), aureusidin 6-glucuronide (Figure 2C), madecassic acid (Figure 2D), pedunculoside (Figure 2E), 6-galloylglucose (Figure 2F), 1,2,6-trigalloyl-β-D-glucopyranose (Figure 2G), propyl gallate (Figure 2H), methyl gallate (Figure 2I), theogallin (Figure 2J), gallic acid (Figure 2K), ellagic acid (Figure 2L), sanguiin H4 (Figure 2M), hamamelitannin (Figure 2N), pyrogallol (Figure 2O), chebulic acid (Figure 2P), chebuloside II (Figure 2Q), and 1,6-bis-O-(3,4,5-trihydroxybenzoyl) hexopyranose (Figure 2R). The scientific literature has documented the presence of gallic acid, ellagic acid, chebulic acid, chebuloside II, methyl gallate, propyl gallate, ethyl gallate, phloroglucinol, pyrogallol, quercetin, kaempferol, and various others compound in the fruit extracts of *T. bellirica* and *T. chebula* [38,39,40]. In our previous study, we conducted qualitative GC-MS headspace analysis on aqueous, methanol and ethyl acetate fruit extracts of *T. bellirica* and *T. chebula*. Our analysis revealed the presence of several notable volatile terpenoids, including eucalyptol, linalool, methoxycitronellal, terpinene-4-ol, camphor, pinocarveol, carvone, endo borneol, L-fenchone, hyscylene, patchoulane, p-cumic aldehyde, and phenylbutanal [11].

Notably, Embaby et al. (2019) showed synergistic antibacterial activity of quinic acid-rich acetone bark extract of *Ficus macrocarpa* var. nitida. with tetracycline against *E. coli* and *S. aureus* [41]. Furthermore, an in-silico molecular docking study revealed that 5-caffeol quinic acid (and other phenolic compounds) may have antibacterial activity due to their efflux pump inhibitory effect. However, the study did not investigate the interactions between the plant extracts and conventional antibiotics. Another study reported the antibacterial activity of quinic acid against *E. coli* and *S. aureus*, with an MIC of 500 µg/mL to 1000 µg/mL, respectively [42]. Additionally, Zhang et al. (2024) reported synergistic antibacterial effects for shikimic acid (625 µg/mL) in combination with penicillin, ampicillin, amoxicillin, and ceftiofur against MRSA, and significantly reduced MIC values reported for the combinations (4 to 16-fold decreases) [43]. Similarly, other investigations have shown that phloroglucinol derivatives exhibited synergistic antibacterial activity against MRSA in combination with vancomycin, penicillin and doxycycline [44,45,46], despite phloroglucinol itself lacking antibacterial activity against MRSA, yielding a high MIC of over 10,000 µg/mL [45]. 

We identified the flavonoid glucuronide, aureusidin 6-glucuronide in the ethyl acetate extracts of *T. bellirica* and *T. chebula*. Notably, studies evaluating the antibacterial activity of this compound are lacking in the literature, as are studies evaluating its effects in combination with conventional antibiotics. In addition, madecassic acid, a pentacyclic triterpenoid has been identified in the *T. chebula* ethyl acetate extracts in our study. Previous studies have reported noteworthy antibacterial activity of madecassic acid against *S. aureus*, MRSA, and *E. coli*, with MIC values of 31.25 (61.9 μM), 62.5 (124 μM), 250 µg/mL (495 μM), respectively [47]. These findings indicate that the madecassic acid exhibits more efficacy against Gram-positive bacteria, including *S. aureus* and MRSA, compared to Gram-negative bacteria such as *E. coli*. This discrepancy in effectiveness may be attributed to variations in cell wall structures and methods of action. These values aid in determining the most effective dosage levels and emphasize the potential of madecassic acid as a specific treatment for antibiotic-resistant bacteria, such as MRSA. Moreover, these findings establish a basis for future investigations, such as carrying out studies with different antibiotics to improve effectiveness. Antibacterial mechanism-focused experiments showed that madecassic acid destroys cell wall integrity, inhibiting the synthesis of soluble proteins and DNA topoisomerase I and II [47]. Pedunculoside, a triterpene saponin has been identified in the methanol and ethyl acetate *T. chebula* extracts. Interestingly, an older study reported the antibacterial effects of pedunculoside against *S. aureus* and *E. coli*, with MIC values of 200 µg/mL [48]. However, there is a shortage of literature on the combined effects of pedunculoside and antibiotics.

Previous work has documented the antibacterial activity of penta-galloyl-glucose against *S. aureus* and *E. coli*, with an MIC of 250 µg/mL (266 µM) against both bacteria [49]. The postulated mechanism of antibacterial activity is related to the suppression of the bacterial type II fatty acid production pathway. Another study reported broad-spectrum antibacterial activity of penta-galloyl-glucose against both methicillin-resistant *S. aureus* and quinolone-resistant *S. aureus* and *E. coli*, with MIC values ranging from 64 to 128 µg/mL (68 µM and 136 µM, respectively) [50]. The antibacterial activities of four derivatives of galloyl-β-D-glucose have also been investigated against multidrug-resistant *E. coli* and *K. pneumoniae* strains, with MIC values from 32 µg/mL to 128 µg/mL, respectively (26–136 µM) [51]. However, combinational interactions of galloyl-β-D-glucose derivatives with reference antibiotics have not been conducted to date.

Our LC/MS experiments also identified the galloyl-glucose derivatives 1,2,6-trigalloyl-β-D-glucopyranose, 1,6-bis-O-(3,4,5-trihydroxybenzoyl) hexopyranose, and 6-O-[(2E)-3-phenyl-2-propenoyl]-1-O-(3,4,5-trihydroxybenzoyl)-β-D-glucopyranose in both *T. bellirica* and *T. chebula* extracts. Previous studies have reported that relatively high levels of gallotannins are present in *T. bellirica* and *T. chebula* extracts [40,52,53,54]. Notably, efflux pump inhibitory activity has previously been demonstrated for 1,2,6-tri-*O*-galloyl-β-D-glucopyranose against multidrug-resistant (MDR) uropathogenic *E. coli* [55]. Additionally, 1,2,6-tri-*O*-galloyl-β-D-glucopyranose exhibits synergistic antibacterial activity in combination with gentamicin and trimethoprim against *E. coli* [56]. Synthetic gallotannins also exhibit antibiofilm and antimicrobial activity against *S. aureus* and MRSA strains [57].

Synergistic interactions between orbifloxacin and propyl gallate against the *E. coli*-resistant strain KVCC 1423 have previously been reported, with MIC values of the combination being reduced from 125 µg/mL (316 µM, orbifloxacin only) to 7.8 µg/mL (19.7 µM), and 312.5 µg/mL (1472 µM, propyl gallate only) to 78 µg/mL (367 µM), respectively [58]. In the present study, through LC-MS analysis, propyl gallate was identified only in the *T. chebula* extracts, although methyl gallate was identified in both *Terminalia* species. Tamang et al. (2022) investigated the antibacterial activities of erythromycin, ampicillin, gentamicin, kanamycin, and ciprofloxacin in combination with gallic acid, methyl gallate, ethyl gallate, propyl gallate, butyl gallate, octyl gallate, dodecyl gallate, and stearyl gallate against MRSA [59]. It was noted in that study that octyl gallate (4 µg/mL) exhibited significant antimicrobial synergy against MRSA in combination with penicillin, ampicillin, cephalothin, gentamicin, tetracycline, erythromycin and lincomycin, reducing their MIC values from 64 µg/mL to 0.25–16 µg/mL. 

A recent study reviewed the antimicrobial characteristics of sanguiins, highlighting their capacity to inhibit bacterial growth and the production of biofilms [60]. The antibacterial activity of sanguiin H6, a closely related compound to sanguiin H4, was demonstrated against *S. aureus* and MRSA, with an MIC value of 250 µg/mL [60,61]. Sanguiin H4 has been identified in the polyphenolic extract of *Sanguisorba officinalis* L., which exhibited antibacterial activity against *S. aureus* and *E. coli* [62]. However, data are not available for the combinatorial interactions of sanguiins with antibiotics to combat AMR. The combination of gallic acid and thiamphenicol, and the combination of hamamelitannin with thiamphenicol or erythromycin, exhibited synergistic antibacterial effects against *E. coli*. (ATCC 25922) [63]. Furthermore, a study detected additional antibacterial interactions against *E. coli* when combining gallic acid with ampicillin, or cefotaxime, and/or marbofloxacin, as well as when combining hamamelitannin with amoxicillin or marbofloxacin. Interestingly, gallic acid and hamamelitannin each have moderate antibacterial activity against *E. coli*, with MIC values of 1024 µg/mL (6024 µM) and 2048 µg/mL (4230 µM), respectively [63]. Bassyouni et al. (2015) noted that hamamelitannin (20 µg/mL) reduces the MIC of vancomycin (4 µg/mL), and clindamycin (32 µg/mL) to 0.25 µg/mL against MRSA strains [64]. Furthermore, hamamelitannin in combination with vancomycin and clindamycin effectively inhibits the biofilm formation in MRSA strains. 

Our present study identified chebulic acid, a gallotannin compound in the aqueous extract of *T. bellirica* and all extracts of *T. chebula.* Chebulic acid has demonstrated numerous biological activities including anti-tumor activity, anti-atherogenic, anti-fibrotic, anti-ulcer, and antioxidant effects [65]. Yang et al. (2020) identified and isolated twenty chebulic acid and brevifolincarboxylic acid derivatives from an ethanolic extract prepared from the arial parts of *Euphorbia hirta* L. [66]. All of those compounds exhibited significant free radical scavenging activities, although antibacterial activity was not investigated in that study. Both ethyl gallate and tri-n-butyl chebulate have been isolated from the aqueous *T. chebula* fruit extract [67]. Both compounds inhibit *K. pneumoniae* growth, with MIC values of 156 µg/mL (787 µM) and 1250 µg/mL, respectively. However, combinational interactions with antibiotics were not investigated in that study. In the present study, chebuloside II was only identified in the methanolic *T. chebula* fruit extract. In contrast, several earlier studies reported the presence of chebuloside II in both *T. bellirica* and *T. chebula* extracts [40,53,54,68]. Chebuloside II-rich *T. chebula* extracts exhibit hepatoprotective effects and prevent liver toxicity in animal models [68], although there is a lack of studies examining the antibacterial activity and combinational interactions of chebuloside II with conventional antibiotics.

In the present study, the extracts were found to be rich in gallic acid, ellagic acid and pyrogallol. Previous studies showed that gallic acid acts synergistically in combination with norfloxacin against *S. aureus* [69]. This combination decreases the MIC of norfloxacin from 156 μg/mL to 49 μg/mL. In addition, gallic acid lowered the MIC of gentamicin against *S. aureus* from 49 μg/mL to 2.5 μg/mL. Similarly, ellagic acid lowered the MIC of tetracycline, chloramphenicol, and tobramycin against a multidrug resistant isolate of *E. coli* [70]. Quave et al. (2012) showed that ellagic acid and its derivatives isolated from the roots of *Rubus ulmifolius* Schott. inhibited *S. aureus* growth by inhibiting biofilm formation [71]. Pyrogallol exhibited antibacterial activity against *S. aureus* and reduced the MIC of norfloxacin from 156 μg/mL to 78 μg/mL, and gentamicin from 49 μg/mL to 2.5 μg/mL [69]. In addition, pyrogallol exhibited broad-spectrum antibacterial activities against methicillin-susceptible *S. aureus*, MRSA, *E. coli* (ATCC 25922), colistin-resistant *E. coli*, and colistin-resistant *K. pneumoniae* [72]. 

We were unable to detect synergistic enhancement of antibacterial activity between *T. bellirica* and *T. chebula* extracts and any of the antibiotics we selected against the bacterial pathogens investigated. However, additive interactions of extracts were noted in some combinations containing penicillin G, amoxicillin, erythromycin, chloramphenicol, tetracycline and ciprofloxacin against *S. aureus*, MRSA, *E. coli*, and ESBL *K. pneumoniae*. This indicates that the phytochemicals present in the *T. bellirica* and *T. chebula* extracts may possess β-lactamase and/or efflux pump inhibitory properties. Further research is required to investigate the effects of these extracts against those resistance mechanisms. 

The toxicity assays using *Artemia* nauplii revealed that all *T. bellirica* and *T. chebula* extracts are nontoxic, thereby indicating their safety as an antimicrobial agent. To determine whether these extracts are suitable for use in medicine, additional testing should be conducted utilizing a panel of mammalian cell lines. Taken together, the findings of our study indicate that *T. bellirica* and *T. chebula* fruit extracts may be a valuable source of antimicrobial compounds for future research and development in the fight against bacterial infections. 

## 4. Materials and Methods

### 4.1. Plant Origins

*Terminalia bellirica* fruit powder (batch no: BNFP/01) was produced by Organic Prime and was purchased online from Navafresh Australia. *Terminalia chebula* fruit powder (batch no: HRP1020) developed by Aarshaveda was obtained online from Sattvic Australia. The traditional Ayurvedic names of *T. bellirica* (Bhitaki, Baheda) and *T. chebula* (Haritaki, Harad) were used to search for the plant herb on the supplier’s website. The provider verified the authenticity and quality of the plant materials. The plant samples were labelled, and voucher specimens NBG-TB0220GU and NBG-TC0220GU for *T. bellirica* and *T. chebula*, respectively, were kept at Griffith University’s Gold Coast campus in the School of Pharmacy and Medical Sciences.

### 4.2. Extract Preparation

After weighing individual 1 g masses of *T. bellirica* and *T. chebula* fruit powders into three separate 50 mL tubes, sterile deionized water, methanol (AR grade), or ethyl acetate (AR grade) were added individually to achieve a total volume of 50 mL [11,34]. The organic solvents (methanol and ethyl acetate) were provided by ChemSupply (Gillman, Australia). Samples were mixed at room temperature for 24 h and then filtered through Whatman No. 54 filter paper (Sigma-Aldrich, Melbourne, Australia) into pre-weighed 50 mL tubes under vacuum pressure. The aqueous samples were lyophilized in an Alpha 1–4 LSC plus benchtop freeze dryer (Martin Christ, Osterode am Harz, Germany) for 72 h to dry the aqueous extracts. The organic solvent samples were subjected to evaporation at a temperature of 40 °C until the evaporation process was fully completed. To calculate the final yields, all dried extracts were weighed, and the mass of extract was determined. The crude extracts were resuspended in 10 mL of 1% dimethyl sulfoxide (DMSO; Merck, Macquarie Park, Australia) and sterilized by passage through 0.22 µm syringe-driven filters (Sarstedt, Mawson Lakes, Australia) and stored at −20 °C until use.

### 4.3. Antibiotics and Bacterial Strains

Powdered antibiotics were purchased from Sigma-Aldrich (Melbourne, Australia), which included penicillin G (potency of 1440–1680 µg/mg), erythromycin (potency ≥850 µg/mg), tetracycline (≥95% purity by HPLC), chloramphenicol (≥98% purity by HPLC), ciprofloxacin (≥98% purity by HPLC), polymyxin B (purity >90%), oxacillin (≥95% purity by TLC), amoxycillin (potency of 900 µg/mg), gentamicin (≥98% purity by HPLC), and vancomycin (potency of ≥900 μg per mg). Antibiotic stock solutions (1 mg/mL) were prepared for broth microdilution assays and stored at −20 °C until required. Oxoid Ltd. (Thebarton, Australia) supplied preloaded standard discs that contained penicillin G (10 IU), erythromycin (10 µg), tetracycline (30 µg), chloramphenicol (30 µg), ciprofloxacin (1 µg), polymyxin B (300IU), oxacillin (1 µg), gentamicin (10 µg), vancomycin (30 µg), Augmentin^®^ (15 µg) and cefoxitin (30 µg). A 10 µL volume of amoxicillin stock solution (0.01 mg/mL) was infused into sterile filter paper discs, which were subsequently placed on Mueller–Hinton (MH) agar and used immediately for disc diffusion assays. Broth microdilution assays were conducted with all reference antibiotics, except for Augmentin^®^ and cefoxitin. 

The American Type Culture Collection (ATCC, Manassas, VA, USA) provided the reference strains of *Escherichia coli* (ATCC 25922), *Staphylococcus aureus* (ATCC 25923), MRSA (ATCC 43300), *Klebsiella pneumoniae* (ATCC 13883), and ESBL *Klebsiella pneumoniae* (ATCC 700603). A clinical isolate strain of ESBL *Escherichia coli* was obtained from the Gold Coast University Hospital in Southport, QLD, Australia and its resistance profile has been previously confirmed by our group [34]. Mueller–Hinton (MH) agar and broth (Oxoid Ltd., Australia) were utilized to cultivate all bacterial strains. The resistance phenotype of the MRSA strain was preserved by culture at 35 °C [73], whilst all other bacterial strains were cultured at 37 °C for 18–24 h. All MH agar plates were prepared in accordance with the instructions provided by the manufacturer and with an agar depth of 4 millimeters.

### 4.4. Antibacterial Susceptibility Screening

The antibacterial activity of all plant extracts in MH agar was investigated using a modified Kirby–Bauer disc diffusion method [34]. In summary, individual colonies isolated from MH agar plates were inoculated in 40 mL MH broth and grown at 37 °C for 18–24 h, except for MRSA, which was grown at 35 °C. The individual bacterial cultures were used to make 0.5 McFarland standards for each strain. Volumes of 100 µL of the 0.5 McFarland standards were spread on fresh MH agar plates. Using sterile forceps, Whatman sterile filter paper discs (6 mm in diameter) were affixed to MH agar, and 10 µL of all extracts resuspended in 1% DMSO was infused into them. Reference antibiotic discs were also tested in this way. The plates were incubated at 37 °C for 18–24 h, except for MRSA, which was incubated at 35 °C. All samples were examined in triplicate. 

The zones of inhibition (ZOIs) were reported as the diameter of the inhibition zones around each disc, which was measured to the nearest whole millimeter (mm) to assess the bacterial growth inhibition. Samples with no visible inhibition were reported to have ZOIs of 6 mm (the diameter of the discs). The ZOI data are presented in the form of bar graphs as the average ± SEM (standard error of mean) of a minimum of three independent studies. One-way analysis of variance (ANOVA) was employed to analyze the differences between the treatment groups and the negative controls. *p*-values of less than 0.01 and 0.001 were deemed to be statistically highly significant and very highly significant, respectively.

### 4.5. Minimum Inhibitory Concentration Determinations

The minimum inhibitory concentration (MIC) values for all extracts and reference antibiotics were determined using a standard 96-well microtiter plate broth microdilution assay [11,34]. A 100 µL volume of the individual extracts and reference antibiotics were added to the top row of the plates. The dilutions were then made down each column of the plates using doubling dilutions. After adding a volume of 100 µL of a 1:100 dilution of 0.5 McFarland cell suspension to every well (except from the sterile controls), the wells were incubated for 20–24 h at 37 °C. After the initial incubation period, a solution (0.4 mg/mL) of p-iodonitrotetrazolium violet (INTZ; Sigma Aldrich, Australia) dye was added to each well on the plate. The plate was then incubated for an additional 2–4 h at room temperature. For determining the MIC values, the lowest concentration of plant extracts or antibiotics that effectively prevented bacterial growth was determined by visual inspection and was indicated by the absence of a red-pink color change. The experiments were conducted in duplicate. MIC values greater than 10,000 μg/mL were classified as inactive, while values between 2000 and 10,000 μg/mL were considered to have low activity. Moderate activity was assigned to MIC values between 1000 and 2000 μg/mL, noteworthy activity to values between 400 and 1000 μg/mL, and good activity to values between 100 and 400 μg/mL. MIC values below 100 μg/mL were regarded as having high activity.

### 4.6. Fractional Inhibitory Concentration Evaluation

Plant extracts and antibiotics that showed antibacterial activity at minimum inhibitory concentrations (MICs) ≤ 3000 µg/mL and ≤2.5 µg/mL, respectively, were selected for combination studies. These were combined in equal proportions (50:50) to study their interactions with susceptible bacterial pathogens. The study examined the interactions between the extracts and antibiotics by calculating the sum of the fractional inhibitory concentrations (ΣFIC) for each combination using the following equations (a = extracts; b = antibiotics):

FIC(a) = MIC (a in combination with b)/MIC (a independently)

FIC(b) = MIC (b in combination with a)/MIC (b independently).

The sum of the fractional inhibitory concentrations (∑FIC) was determined by applying the formula ∑FIC = FIC(a) + FIC(b).

The interactions were classified as synergistic (∑FIC ≤ 0.5), additive (∑FIC > 0.5–≤ 1.0), indifferent (∑FIC > 1.0–≤ 4.0), or antagonistic (∑FIC > 4.0) [74].

### 4.7. Toxicity Assays

The toxicity of the plant extracts and controls were assessed using standard *Artemia franciscana* Kellogg nauplii lethality assays (ALA) [11]. In summary, 400 µL of plant extracts (2 mg/mL concentration, diluted in artificial seawater) and 400 µL of artificial seawater with newly hatched (within 1 day) *A. franciscana* nauplii were added separately in each well of a 48-well plate. On all plates, a 400 µL volume of negative control (32 g/L artificial seawater; Red Sea) and positive control (1 mg/mL sodium azide) was added. The plates were then incubated at 25 °C ± 1 °C for 24 h, and the number of live shrimps was determined. Probit analysis was employed to determine the LC_50_ values, which represent the concentration of extract or control required to cause mortality in 50% of the *A. franciscana* nauplii in separate wells. The LC_50_ values were calculated graphically using the mean percentage of three repeated experiments, representing the concentration that resulted in 50% mortality.

### 4.8. Non-Targeted Headspace LC-MS for Quantitative Analysis

A Vanquish Ultra High-Performance Liquid Chromatography (UHPLC) system (Thermo Fisher Scientific, Waltham, MA, USA) was utilized to conduct a non-targeted headspace metabolic profile analysis on all samples [34]. The separation of compounds was achieved using an Accucore ™ RP-MS column (100 mm × 2.1 mm) with a particle size of 2.6 μm, which was linked to an Orbitrap Exploris 120 mass spectrometer (Thermo Fisher Scientific). The UHPLC system was fitted with a quaternary pump at a flow rate of 0.6 mL/min. The separation used the following mobile phases: (A) a solution of 0.1% *v*/*v* formic acid in ultrapure water and (B) acetonitrile (MeCN) solution containing 0.1% *v*/*v* formic acid. The Xcalibur acquisition software (version 2.0) was utilized to create a gradient flow for compound separation. The flow consisted of the following steps, with a total duration of 24 min: 5% B for 5 min, a linear increase from 5% to 30% B over 5 min, 30% B for 3 min, a linear increase from 30% to 90% B over 4 min, isocratic elution at 90% B for 4 min to flush the column, and a linear decrease from 90% to 5% B over 1 min. The column was re-equilibrated by running an isocratic separation at 5% B of the mobile phase for a duration of 2 min between each separation. To obtain chromatogram peaks of high quality, plant extract samples were analyzed at three distinct concentrations (0.25 mg/mL, 0.5 mg/mL, and 1 mg/mL) using the Vanquish HPLC system (Thermo Fisher Scientific, Waltham, MA, USA) linked to the same column as mentioned above. Plant samples exhibited enhanced chromatogram peaks of superior quality when used at a concentration of 1 mg/mL. 

The mass spectra of the eluted compounds were examined using the Orbitrap Exploris 120 mass spectrometer in the information-dependent acquisition (IDA) mode. The Orbitrap system employed electrospray ionization (ESI) in the negative ionization mode, utilizing specified settings including a vaporizer temperature of 350 °C, sheath gas pressure of 60 psi, auxiliary gas pressure of 15 psi, sweep gas pressure of 2 psi, and a spray voltage ranging from 2.5 KV to 5 KV. The data were analyzed using Compound Discoverer^TM^ software 3.3. The identification of the unknown natural products utilized local database searches and online statistical analysis. To remove the background and differentiate individual components of the extract, the data files for each extract were analyzed individually and compared to the blank file. In the Compound Discoverer function, result filters were employed to utilize databases such as Mz cloud, ChemSpider, Predicted Compositions, and MassList to achieve either a partial or complete match of the compounds that were discovered. The Compound Discoverer software 3.3 was utilized to generate Excel files containing the discovered compounds from each extract. The files were subsequently analyzed to generate a catalogue containing potential putative compounds. Following the consolidation of duplicates by pivot table analysis in Microsoft Excel, the relative abundance was calculated as a percentage of the total area.

## 5. Conclusions

Due to increasing levels of antibiotic-resistant bacterial strains, there is a pressing demand for new antibacterial medications, which has generated interest in natural products as potential sources. The results of our study demonstrate that the active extracts derived from *Terminalia bellirica* and *Terminalia chebula* can effectively hinder the growth of both resistant and susceptible strains of bacteria. This suggests that the extracts possess unique antibacterial mechanisms that require further examination. In addition, all extracts enhanced the effectiveness of various conventional antibiotics, specifically β-lactam antibiotics and tetracycline. This may allow the reactivation of these antibiotics, even in bacterial strains that are otherwise resistant to their actions. The method of potentiation has not been discovered yet, although it is probable that the components of the extract may deactivate bacterial extended spectrum β-lactamase enzymes and efflux pumps, leading to an increase in the concentration of antibiotics inside the cells. Future studies are required to confirm these mechanisms, as well as to test other possible potentiation pathways. Several phytochemicals that were identified in the extracts may contribute to these actions, suggesting that they could be valuable targets for new antibacterial agent development, although further confirmation is required. Subsequent studies should prioritize the isolation of these compounds and examine their capacity to augment the effectiveness of existing antibiotics. Also, nuclear magnetic resonance (NMR) is essential for the conclusive structural identification of the components present in the extract.

## Figures and Tables

**Table 1:** MIC values (µg/mL) for aqueous, methanol, and ethyl acetate of *T. bellirica* and *T. chebula* extracts, and the reference (positive control) antibiotics against the six bacterial strains.

Extract Type or Antibiotic	Bacterial Species and MIC (µg/mL)					
	*S. aureus*	MRSA	*E. coli*	ESBL *E. coli*	*K. pneumoniae*	ESBL *K. pneumoniae*
TB-AQ	392	196	**3138**	1569	784	**3138**
TB-MeOH	94	189	755	755	1509	755
TB-EtOAc	900	900	Inactive	Inactive	Inactive	Inactive
TCh-AQ	139	139	**2225**	**2225**	556	**2225**
TCh-MeOH	189	189	**3019**	1509	1509	1509
TCh-EtOAc	306	306	Inactive	Inactive	1225	Inactive
PENG	**1.25**	>2.5	>2.5	>2.5	>2.5	>2.5
ERY	**0.31**	>2.5	>2.5	>2.5	>2.5	>2.5
TET	**0.16**	**0.04**	**0.31**	>2.5	**0.625**	>2.5
CHL	>2.5	>2.5	**2.5**	>2.5	**2.5**	>2.5
CIP	**0.16**	**0.625**	**0.02**	>2.5	**0.02**	**0.16**
POLB	>2.5	>2.5	**0.02**	**0.02**	**0.02**	**0.04**
OXA	**0.16**	>2.5	>2.5	>2.5	>2.5	>2.5
AMX	**0.625**	>2.5	>2.5	>2.5	>2.5	>2.5
GEN	>2.5	>2.5	**0.625**	>2.5	**0.625**	>2.5

MIC values for extracts with no antibacterial activity (Inactive; MIC > 10,000 µg/mL), low activity (2000–5000 µg/mL; shown in bold), moderate activity (1000–2000 µg/mL; shown in blue), noteworthy activity (400–1000 µg/mL; shown in red), or good activity (100–400 µg/mL; shown in green). Reference antibiotics: PENG = penicillin G, ERY = erythromycin, TET = tetracycline, CHL = chloramphenicol, CIP = ciprofloxacin, POLB = polymyxin B, OXA = oxacillin, AMX = amoxycillin, GEN = gentamicin, and VAN = vancomycin. Active antibiotics are shown in bold, while lack of antibiotic activity was observed when MIC values > 2.5 µg/mL.

**Table 2:** ∑FIC values calculated for combinations of T. bellirica and T. chebula extracts and conventional antibiotics.

Bacteria	Extracts	PENG	ERY	TET	CHL	CIP	POLB	OXA	AMX	GEN	VAN
***S. aureus***	TB-AQ	**0.53**	1.26	1.50	-	1.50	-	1.25	**0.56**	-	1.06
	TB-MeOH	1.02	2.13	1.13	-	2.25	-	1.25	1.03	-	2.03
	TB-EtOAc	**0.75**	1.13	1.06	-	2.06	-	2.13	1.25	-	1.50
	TChAQ	1.06	1.25	**0.75**	-	1.50	-	1.50	1.13	-	1.06
	TCh-MeOH	1.03	1.13	1.25	-	1.25	-	1.25	1.06	-	1.03
	TCh-EtOH	**0.75**	**0.75**	1.25	-	2.50	-	2.50	**1.00**	-	1.50
**MRSA**	TB-AQ	-	-	**1.00**	-	1.06	-	-	-	-	1.03
	TB-MeOH	-	-	1.50	-	1.03	-	-	-	-	1.02
	TB-EtOAc	-	-	1.02	-	**0.63**	-	-	-	-	1.50
	TCh-AQ	-	-	**1.00**	-	2.13	-	-	-	-	2.06
	TCh-MeOH	-	-	**1.00**	-	1.06	-	-	-	-	1.03
	TCh-EtOAc	-	-	1.06	-	**1.00**	-	-	-	-	1.50
***E. coli***	TB-AQ	-	-	1.50	1.25	2.25	**64.50**	-	-	2.00	-
	TB-MeOH	-	-	1.52	1.06	**4.00**	**17.50**	-	-	1.25	-
	TCh-AQ	-	-	1.50	1.25	2.25	**64.50**	-	-	2.00	-
	TCh-MeOH	-	-	**0.76**	1.25	2.25	**32.25**	-	-	2.00	-
**ESBL *E. coli***	TB-AQ	-	-	-	-	-	**16.50**	-	-	-	-
	TB-MeOH	-	-	-	-	-	**5.00**	-	-	-	-
	TCh-AQ	-	-	-	-	-	**32.50**	-	-	-	-
	TCh-MeOH	-	-	-	-	-	**16.50**	-	-	-	-
***K. pneumoniae***	TB-AQ	-	-	1.25	1.06	2.00	**9.00**	-	-	1.25	-
	TB-MeOH	-	-	1.50	1.12	3.00	**16.50**	-	-	1.50	-
	TB-EtOAc	-	-	-	-	-	-	-	-	-	-
	TCh-AQ	-	-	1.25	1.06	**4.00**	**9.00**	-	-	1.25	-
	TCh-MeOH	-	-	**0.75**	**0.56**	3.00	**16.50**	-	-	1.50	-
	TCh-EtOAc	-	-	**0.63**	**1.00**	2.12	**63.00**	-	-	**4.00**	-
**ESBL *K. pneumoniae***	TB-AQ	-	-	-	-	1.25	**8.25**	-	-	-	-
	TB-MeOH	-	-	-	-	**1.00**	2.50	-	-	-	-
	TCh-AQ	-	-	-	-	2.50	**16.60**	-	-	-	-
	TCh-MeOH	-	-	-	-	1.50	**8.75**	-	-	-	-

∑ FIC values of aqueous (TB-AQ, TCh-AQ), methanolic (TB-MeOH, TCh-MeOH) and ethyl acetate (TB-EtOAc, TCh-EtOAc) extracts of *T. bellirica* and *T. chebula*, respectively, in combination with reference antibiotics against antibiotic-sensitive and antibiotic-resistant strains of *S. aureus*, MRSA, *E. coli*, ESBL *E. coli*, *K. pneumoniae*, and ESBL *K. pneumoniae*. **Additive interaction: >0.5 ≤ 1.00** and are in blue color, indifferent interaction: >1.01–≤4.00, antagonistic interaction: >4.0 and are in red color.—indicates the extract and/or the antibiotic was inactive against the bacteria being tested. Reference antibiotics: PENG = penicillin G, ERY = erythromycin, TET = tetracycline, CHL = chloramphenicol, CIP = ciprofloxacin, POL B = polymyxin B, OXA = oxacillin, AMX = amoxycillin, GEN = gentamicin, and VAN = vancomycin.

**Table 3:** LC-MS putative identification and % relative abundance of phytochemicals identified in the fruit extracts of *T. bellirica* (TB) and *T. chebula* (TCh).

Retention Time (min)	Molecular Weight	Empirical Formula	Putative Compounds	% Relative Abundance (TB)			% Relative Abundance (TCh)		
				AQ	MeOH	EtOAc	AQ	MeOH	EtOAc
1.496	192.06305	C_7_H_12_O_6_	Quinic acid	3.13%	0.33%	0.19%	0.39%	-	1.53%
1.573	344.07409	C_14_H_16_O_10_	Theogallin	-	3.47%	-	-	0.03%	0.02%
1.784	302.06328	C_12_H_14_O_9_	Pyrogallol-2-O-glucuronide	-	0.21%	-	0.07%	-	-
1.793	244.0581	C_10_H_12_O_7_	1-O-Galloylglycerol	-	-	0.02%	-	-	-
1.905	236.0684	C_12_H_12_O_5_	Dillapional	0.01%	-	-	-	T	0.01%
1.923	316.0793	C_13_H_16_O_9_	Ginnalin B	0.01%	-	-	-	-	-
1.975	149.08397	C_9_H_11_NO	Venoterpine	-	-	0.05%	0.04%	-	-
2.045	294.03749	C_13_H_10_O_8_	Banksiamarin B	-	-	0.15%	0.17%	0.05%	0.13%
2.058	174.05268	C_7_H_10_O_5_	Shikimic acid	-	0.41%	0.67%	2.64%	3.14%	4.80%
2.237	154.02646	C_7_H_6_O_4_	Gentisic acid	0.02%	0.05%	-	0.01%	-	0.09%
2.296	448.15769	C_19_H_28_O_12_	8-O-Acetyl shanzhiside methyl ester	-	-	-	0.19%	0.18%	-
2.356	332.07411	C_13_H_16_O_10_	6-Galloylglucose	6.06%	3.97%	3.39%	3.74%	0.68%	-
2.444	448.06386	C_20_H_16_O_12_	Ellagic acid 2-rhamnoside	0.11%	1.44%	0.69%	0.01%	0.21%	0.33%
2.51	292.02177	C_13_H_8_O_8_	Brevifolincarboxylic acid	0.09%	-	-	0.11%	0.03%	0.61%
2.56	288.0844	C_12_H_16_O_8_	Phlorin	-	0.02%	-	0.03%	0.02%	-
3.11	484.0848	C_20_H_20_O_14_	Gallic acid 3-O-(6-galloylglucoside)	0.74%	-	-	-	-	-
3.158	212.06824	C_10_H_12_O_5_	Propyl gallate	-	-	-	0.30%	0.35%	0.12%
4.4	277.05821	C_13_H_11_NO_6_	Salfredin C1	-	-	-	0.14%	-	0.03%
4.847	184.03703	C_8_H_8_O_5_	Methyl gallate	0.25%	11.39%	-	0.04%	1.05%	-
5.638	444.19969	C_21_H_32_O_10_	Cynaroside A	-	-	-	0.02%	0.02%	-
8.848	636.09627	C_27_H_24_O_18_	1,3,4-Trigalloyl-β-D-glucopyranose	-	-	-	-	0.07%	0.25%
8.44	484.0855	C_20_H_20_O_14_	1,6-Bis-O-(3,4,5-trihydroxybenzoyl) hexopyranose	5.13%	5.19%	2.52%	6.52%	2.16%	3.78%
8.961	478.07436	C_21_H_18_O_13_	Quercetin 7-glucuronide	-	-	-	-	-	0.49%
8.985	478.0743	C_21_H_18_O_13_	Quercetin 3-O-glucuronide	-	0.08%	-	-	-	-
9.316	470.01176	C_21_H_10_O_13_	Sanguisorbic acid dilactone	-	0.08%	-	0.56%	0.61%	0.19%
9.567	634.08082	C_27_H_22_O_18_	Sanguiin H4	0.84%	-	0.07%	-	5.63%	0.54%
9.811	478.07452	C_21_H_18_O_13_	Miquelianin	0.04%	0.42%	-	0.37%	0.60%	0.39%
9.902	152.04703	C_8_H_8_O_3_	Vanillin	-	-	-	0.16%	0.17%	0.17%
9.921	126.03146	C_6_H_6_O_3_	Phloroglucinol	0.10%	1.93%	-	0.07%	0.24%	0.27%
10.043	636.09614	C_27_H_24_O_18_	1,2,6-Trigalloyl-β-D-glucopyranose	0.09%	5.44%	2.85%	0.11%	1.42%	2.22%
10.158	634.08037	C_27_H_22_O_18_	Corilagin	-	-	-	-	0.15%	-
10.357	524.1533	C_24_H_28_O_13_	Barbatoflavan	0.01%	-	-	-	-	-
10.419	484.08522	C_20_H_20_O_14_	Hamamelitannin	2.10%	0.02%	0.62%	0.02%	0.11%	-
10.421	126.0316	C_6_H_6_O_3_	Pyrogallol	6.28%	5.28%	2.19%	6.29%	3.82%	4.87%
10.422	296.05248	C_13_H_12_O_8_	cis-Coutaric acid	0.33%	0.86%	0.19%	0.31%	0.93%	1.05%
10.476	636.09612	C_27_H_24_O_18_	1,4,6-Trigalloyl-β-D-glucopyranose	-	-	-	-	-	0.63%
10.55	601.99658	C_28_H_10_O_16_	Diellagilactone	-	-	-	1.13%	0.60%	-
10.703	372.1055	C_16_H_20_O_10_	Veranisatin C	0.02%	0.04%	-	-	-	-
10.754	636.09608	C_27_H_24_O_18_	1,3,6-Tri-O-galloyl-β-D-glucose	-	0.25%	0.93%	0.64%	-	-
11.305	170.02138	C_7_H_6_O_5_	Phloroglucinic acid	0.98%	2.80%	4.33%	0.98%	0.83%	1.24%
11.091	610.1535	C_27_H_30_O_16_	Rutin	-	0.14%	0.10%	-	0.03%	0.08%
11.176	432.10539	C_21_H_20_O_10_	Vitexin	-	-	-	-	0.05%	-
11.257	304.0579	C_15_H_12_O_7_	Nigrescin	T	0.02%	-	-	-	-
11.272	610.1527	C_27_H_30_O_16_	Quercetin 3-O-rhamnoside-7-O-glucoside	0.05%	-	-	-	-	-
11.448	302.04213	C_15_H_10_O_7_	Quercetin	0.11%	0.33%	0.17%	0.02%	-	-
11.473	464.0951	C_21_H_20_O_12_	Myricitrin	0.05%	0.13%	0.19%	-	-	-
11.529	422.0846	C_19_H_18_O_11_	1,5,8-Trihydroxy-9-oxo-9H-xanthen-3-yl β-D-glucopyranoside	0.02%	0.05%	-	-	-	-
11.589	170.02147	C_7_H_6_O_5_	Gallic acid	26.23%	8.64%	2.60%	23.90%	1.90%	1.56%
11.756	216.0994	C_10_H_16_O_5_	(4S,5S,8S,10R)-4,5,8-trihydroxy-10-methyl-3,4,5,8,9,10-hexahydro-2H-oxecin-2-one	0.05%	-	-	0.06%	-	1.23%
11.822	462.07961	C_21_H_18_O_12_	Aureusidin 6-glucuronide	-	-	2.52%	-	-	0.04%
11.905	286.04729	C_15_H_10_O_6_	Maritimetin	-	-	-	T	0.01%	0.01%
11.906	594.15829	C_27_H_30_O_15_	Palasitrin	-	-	-	0.01%	-	0.03%
11.938	176.04708	C_10_H_8_O_3_	4-Methylumbelliferone hydrate	-	-	-	0.03%	0.03%	-
11.953	310.10477	C_15_H_18_O_7_	(E)-1-O-Cinnamoyl-β-D-glucose	-	-	-	-	-	1.67%
12.141	584.1169	C_28_H_24_O_14_	2″-O-Galloylisovitexin	-	-	-	-	-	0.01%
12.183	448.0996	C_21_H_20_O_11_	Maritimein	-	0.01%	0.02%	-	-	-
12.185	302.00627	C_14_H_6_O_8_	Ellagic acid	2.45%	10.74%	33.49%	8.39%	10.46%	11.96%
12.201	220.07336	C_12_H_12_O_4_	Eugenitin	-	-	-	-	-	0.04%
12.233	262.0476	C_13_H_10_O_6_	Maclurin	-	0.02%	-	0.04%	-	-
12.254	148.05223	C_9_H_8_O_2_	trans-Cinnamic acid	-	-	-	0.76%	0.96%	0.87%
12.38	334.0325	C_15_H_10_O_9_	3,5,6,7,2′,3′,4′-Heptahydroxyflavone	-	0.20%	-	-	-	-
12.503	192.07847	C_11_H_12_O_3_	(R)-Shinanolone	-	-	-	0.04%	0.03%	0.05%
12.504	310.10483	C_15_H_18_O_7_	(2S,3R,4S,5S,6R)-3,4,5-trihydroxy-6-(hydroxymethyl) oxan-2-yl (2E)-3-phenylprop-2-enoate	-	-	-	2.25%	-	-
12.509	610.1894	C_28_H_34_O_15_	Neohesperidin	-	-	0.16%	-	-	-
12.584	498.1741	C_23_H_30_O_12_	Eucaglobulin	-	0.02%	-	-	-	-
12.593	436.13688	C_21_H_24_O_10_	Nothofagin	-	-	-	-	-	0.12%
12.721	680.37692	C_36_H_56_O_12_	Tenuifolin	-	-	-	-	0.11%	0.37%
12.8	486.33397	C_30_H_46_O_5_	Bassic acid	-	-	-	-	-	0.05%
12.844	518.1785	C_26_H_30_O_11_	Phellodensin E	-	-	0.05%	-	-	-
12.889	346.1052	C_18_H_18_O_7_	Hamilcone	-	-	-	0.02%	-	0.01%
12.914	190.13544	C_13_H_18_O	β-Damascenone	-	-	-	-	0.02%	0.06%
12.915	666.39739	C_36_H_58_O_11_	Chebuloside II	-	-	-	-	3.85%	-
12.924	302.1152	C_17_H_18_O_5_	Lusianin	0.01%	-	-	-	-	-
12.929	356.03763	C_14_H_12_O_11_	(+)-Chebulic acid	0.54%	-	-	3.98%	1.40%	2.88%
12.967	330.14651	C_19_H_22_O_5_	Hericenone A	-	-	-	-	0.03%	-
12.969	504.34417	C_30_H_48_O_6_	Madecassic acid	-	-	-	-	-	1.42%
12.971	202.13522	C_14_H_18_O	(±)-Anisoxide	-	-	-	-	-	0.05%
12.973	244.14583	C_16_H_20_O_2_	Lahorenoic acid C	-	-	-	-	-	0.02%
13.208	288.06318	C_15_H_12_O_6_	Eriodictyol	-	-	-	-	-	0.27%
13.354	314.1154	C_18_H_18_O_5_	Crotaoprostrin	-	-	-	-	-	0.01%
13.428	442.1992	C_25_H_30_O_7_	Exiguaflavanone M	-	-	0.13%	-	-	-
13.443	460.13716	C_23_H_24_O_10_	7-Hydroxy-5,6-dimethoxyflavone 7-glucoside	-	-	-	-	0.01%	-
13.451	274.08371	C_15_H_14_O_5_	Phloretin	-	-	-	0.01%	0.02%	0.01%
13.536	462.11641	C_22_H_22_O_11_	Leptosin	-	0.01%	-	0.01%	0.06%	-
13.59	302.04255	C_15_H_10_O_7_	Bracteatin	-	-	-	0.01%	0.01%	-
13.592	450.07961	C_20_H_18_O_12_	Quercetin 4′-galactoside	-	-	-	0.01%	-	-
13.593	502.1836	C_26_H_30_O_10_	Flavaprin	-	-	0.07%	-	-	-
13.642	514.18403	C_27_H_30_O_10_	Baohuoside 1	-	-	-	T	0.01%	-
13.678	568.12178	C_28_H_24_O_13_	Isoorientin 2″-p-hydroxybenzoate	-	-	-	-	-	0.04%
13.703	280.13067	C_15_H_20_O_5_	Artabsinolide A	-	-	-	-	-	0.02%
13.725	330.0374	C_16_H_10_O_8_	Blighinone	0.12%	-	-	-	-	-
13.805	444.10499	C_22_H_20_O_10_	3′-O-Methylderhamnosylmaysin	-	-	-	0.04%	0.12%	0.08%
13.805	292.09447	C_15_H_16_O_6_	(S)-Angelicain	-	-	-	0.05%	-	-
13.806	462.11629	C_22_H_22_O_11_	6-O-[(2E)-3-Phenyl-2-propenoyl]-1-O-(3,4,5-trihydroxybenzoyl)-β-D-glucopyranose	-	-	-	0.08%	1.02%	0.18%
14.024	500.1674	C_26_H_28_O_10_	Ikarisoside A	-	-	0.03%	-	-	-
14.078	534.28285	C_29_H_42_O_9_	Corchoroside A	-	-	-	-	-	0.02%
14.358	272.06832	C_15_H_12_O_5_	Naringenin	-	-	-	-	-	0.18%
14.367	470.33907	C_30_H_46_O_4_	Glycyrrhetinic acid	-	-	-	-	0.08%	0.01%
14.561	226.1203	C_12_H_18_O_4_	Allixin	-	-	0.03%	-	-	-
14.783	234.16167	C_15_H_22_O_2_	Valerenic acid	-	-	-	-	-	0.13%
14.928	202.17175	C_15_H_22_	Rulepidadiene B	-	-	-	-	-	0.08%
14.93	222.16153	C_14_H_22_O_2_	Rishitin	-	-	-	-	-	0.04%
15.046	426.09477	C_22_H_18_O_9_	Epiafzelechin 3-O-gallate	-	-	-	-	-	0.01%
15.454	650.4026	C_36_H_58_O_10_	Pedunculoside	-	-	-	-	1.18%	2.61%
16.257	738.41926	C_39_H_62_O_13_	Isonuatigenin 3-[rhamnosyl-(1->2)-glucoside]	-	-	-	-	0.04%	0.12%
16.301	470.1941	C_26_H_30_O_8_	Limonin	-	-	0.14%	-	-	-
16.478	540.1663	C_25_H_32_O_11_S	Sumalarin B	-	-	0.02%	-	-	-
16.532	316.1308	C_18_H_20_O_5_	Methylodoratol	-	-	0.02%	-	-	-
16.787	472.2097	C_26_H_32_O_8_	Kushenol H	-	-	0.07%	-	-	-
16.844	344.0532	C_17_H_12_O_8_	3,4,3′-Tri-O-methylellagic acid	-	-	0.01%	-	-	-
16.876	252.20856	C_16_H_28_O_2_	Isoambrettolide	-	-	-	-	-	0.32%
16.889	300.1361	C_18_H_20_O_4_	Angoletin	-	-	T	-	-	-
17.064	440.1828	C_25_H_28_O_7_	Lonchocarpol E	-	-	0.15%	-	-	-
17.209	544.2668	C_30_H_40_O_9_	Physagulin F	-	-	0.10%	-	-	-
17.275	342.14638	C_20_H_22_O_5_	Brosimacutin C	-	-	-	-	T	-
17.276	180.11471	C_11_H_16_O_2_	Jasmolone	-	0.61%	1.48%	0.05%	-	-
17.31	504.34409	C_30_H_48_O_6_	Protobassic acid	-	-	-	1.26%	0.02%	0.01%
17.33	468.32326	C_30_H_44_O_4_	Glabrolide	-	-	-	0.87%	-	3.19%
17.33	502.32964	C_30_H_46_O_6_	Medicagenic acid	-	-	-	-	T	0.07%
17.331	200.15605	C_15_H_20_	(S)-gamma-Calacorene	-	-	-	0.02%	-	0.29%
17.363	696.40877	C_37_H_60_O_12_	Momordicoside E	-	-	-	-	0.01%	0.04%
17.626	286.08357	C_16_H_14_O_5_	Homobutein	-	T	0.01%	-	-	0.01%
17.635	282.12541	C_18_H_18_O_3_	Ohobanin	0.01%	T	0.09%	T	-	0.06%
17.671	652.27302	C_32_H_44_O_14_	Dicrocin	-	-	-	-	-	0.03%
17.902	424.1881	C_25_H_28_O_6_	Paratocarpin G	T	-	-	-	-	-
17.949	456.2144	C_26_H_32_O_7_	Antiarone J	-	0.54%	-	-	-	-
18.124	372.2509	C_20_H_36_O_6_	Sterebin Q4	-	-	0.01%	-	-	-
18.164	328.1309	C_19_H_20_O_5_	2′,3′,4′,6′-Tetrameth oxychalcone	-	-	0.05%	-	-	-
18.237	428.1831	C_24_H_28_O_7_	Heteroflavanone B	-	-	0.05%	-	-	-
18.237	312.13615	C_19_H_20_O_4_	Desmosdumotin C	T	-	0.02%	T	-	0.02%
18.292	546.35535	C_32_H_50_O_7_	Hovenidulcigenin B	-	-	-	0.04%	-	-
18.339	236.1776	C_15_H_24_O_2_	Capsidiol	-	-	-	-	-	0.02%
18.346	268.13069	C_14_H_20_O_5_	Kamahine C	0.02%	0.01%	-	-	0.01%	0.15%
18.363	336.0994	C_20_H_16_O_5_	Ciliatin A	-	-	-	-	0.01%	-
18.47	466.1989	C_27_H_30_O_7_	Eriotriochin	-	0.50%	-	-	-	-
18.498	340.09447	C_19_H_16_O_6_	Ambanol	-	-	-	-	0.01%	-
18.507	484.31803	C_30_H_44_O_5_	Liquoric acid	-	-	-	T	0.01%	0.04%
18.623	488.35017	C_30_H_48_O_5_	Pitheduloside I	-	-	-	T	0.06%	0.11%
19.641	226.09903	C_15_H_14_O_2_	7-Hydroxyflavan	-	0.84%	-	0.03%	-	0.05%
18.748	208.1096	C_12_H_16_O_3_	Isoelemicin	0.01%	-	0.19%	-	-	-
18.767	526.2565	C_30_H_38_O_8_	Kosamol A	-	0.52%	-	-	-	-
18.824	300.0997	C_17_H_16_O_5_	2′,4′-Dihydroxy-3,4-dimethoxychalcone	-	-	0.07%	-	-	-
18.987	372.1208	C_20_H_20_O_7_	Tangeretin	-	-	0.03%	-	-	-

Compounds identified in both plant extracts are in black; compounds identified only in *T. bellirica* (TB) extracts are in red; compounds identified only in *T. chebula* (TCh) extracts are in green; and compounds less than 0.01% of the total area were considered as trace amounts and are denoted as T.

## Data Availability

Data are either presented within the manuscript or are available from the corresponding author upon reasonable request.

## Supplementary Materials

The following supporting information can be downloaded at: https://www.mdpi.com/article/10.3390/antibiotics13100994/s1, Figure S1: LC-MS total compound chromatograms of (**A**) TB-Aq (*Terminalia bellirica* aqueous extract), (**B**) TB-MeOH (*Terminalia bellirica* methanolic extract), and (**C**) TB-EtOAc (*Terminalia bellirica* ethyl acetate extract). Figure S2: LC-MS total compound chromatograms of (**A**) TCh-Aq (*Terminalia chebula* aqueous extract), (**B**) TB-MeOH (*Terminalia chebula* methanolic extract), and (**C**) TB-EtOAc (*Terminalia chebula* ethyl acetate extract). Table S1: LC-MS putative identification and % relative abundance of phytochemicals identified in the extracts of *Terminalia bellirica*. Compounds less than 0.01% of the total area were considered as trace amounts and denoted as T. Table S2: LC-MS putative identification and % relative abundance of phytochemicals identified in the extracts of *Terminalia chebula*. Compounds less than 0.01% of the total area were considered as trace amounts and denoted as T.
