# Supplementary material for: Combinations of Terminalia bellirica (Gaertn.) Roxb. and Terminalia chebula Retz. Extracts with Selected Antibiotics Against Antibiotic-Resistant Bacteria: Bioactivity and Phytochemistry
Source: Antibiotics (Basel). 2024 Oct 19;13(10):994. doi: 10.3390/antibiotics13100994 (PMC11504310; doi:10.3390/antibiotics13100994)
Supplement: Supplementary file 1 [file antibiotics-13-00994-s001.zip › antibiotics-3241352-supplementary.pdf]

Electronic Supplementary Material

Combinations of *Terminalia bellirica* (Gaertn.) Roxb. and *Terminalia chebula* Retz. Extracts with Selected Antibiotics Against Antibiotic-Resistant Bacteria: Bioactivity and Phytochemistry

Gagan Tiwana [1], Ian Edwin Cock [2] and Matthew James Cheesman [1,*]

[1] School of Pharmacy and Medical Sciences, Gold Coast Campus, Griffith University, Gold Coast 4222, Australia; g.tiwana@griffith.edu.au
[2] School of Environment and Science, Nathan Campus, Griffith University, Brisbane 4111, Australia; i.cock@griffith.edu.au
* Correspondence: m.cheesman@griffith.edu.au; Tel.: +61-7-55529230

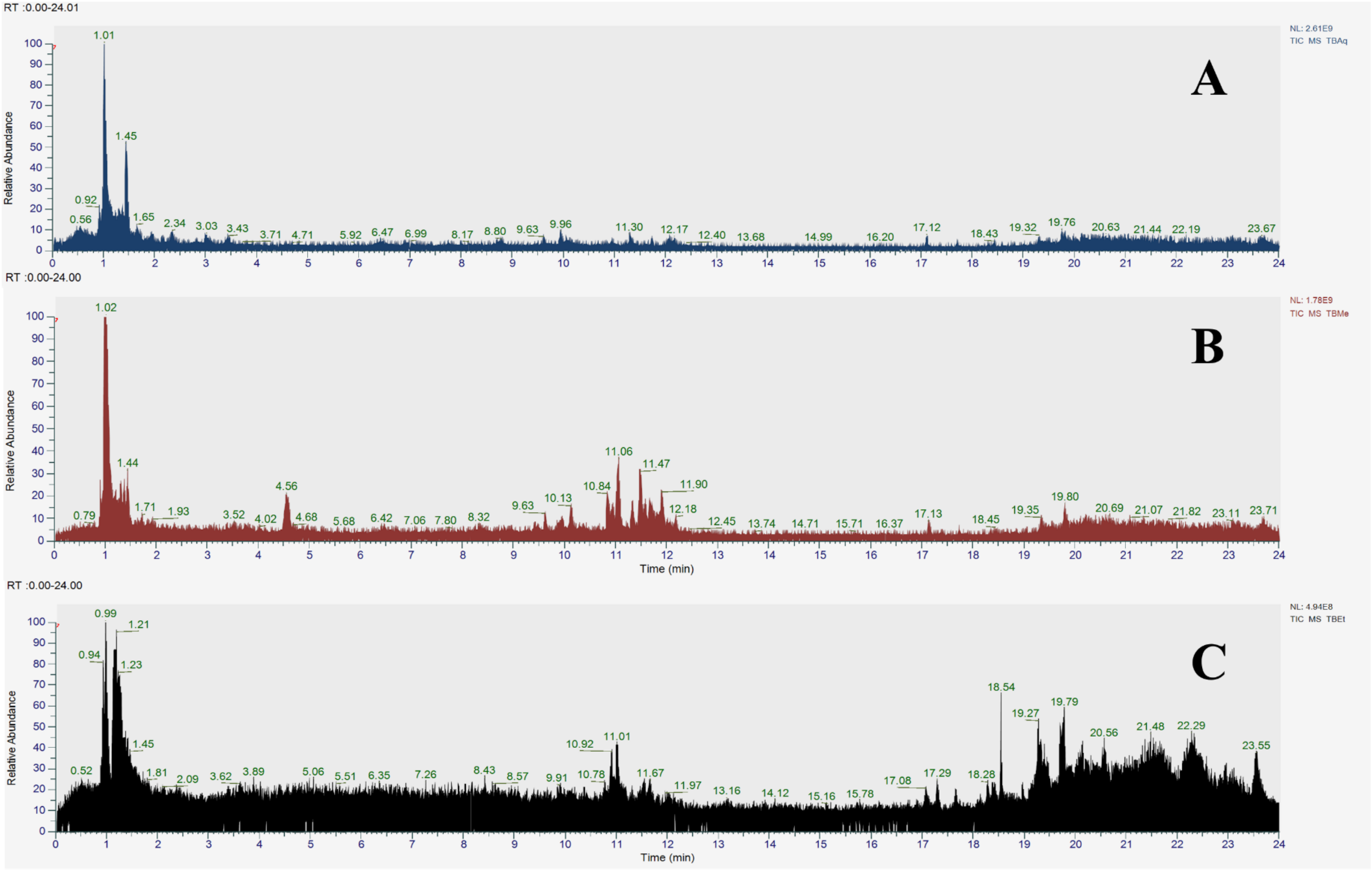

Figure S1: LC-MS total compound chromatograms of (A) TB-Aq (*Terminalia bellirica* aqueous extract), (B) TB-MeOH (*Terminalia bellirica* methanolic extract), and (C) TB-EtOAc (*Terminalia bellirica* ethyl acetate extract).

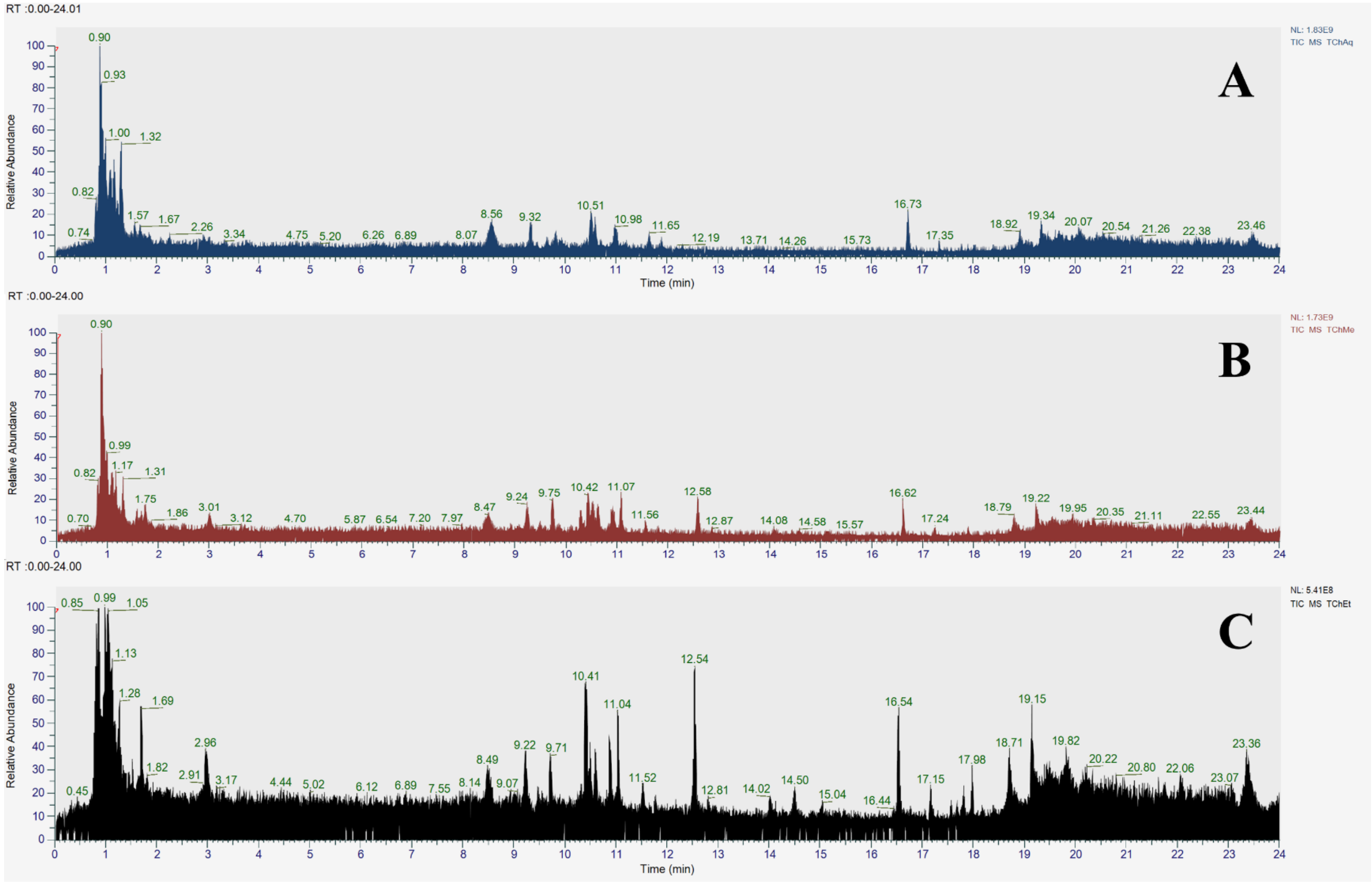

Figure S2: LC-MS total compound chromatograms of (**A**) TCh-Aq (*Terminalia chebula* aqueous extract), (**B**) TB-MeOH (*Terminalia chebula* methanolic extract), and (**C**) TB-EtOAc (*Terminalia chebula* ethyl acetate extract).

Table S1: LC-MS putative identification and % relative abundance of phytochemicals identified in the extracts of *Terminalia bellirica*. Compounds less than 0.01% of the total area were considered as trace amounts and denoted as T.

Retention time [min]	Molecular weight	Empirical Formula	Putative Compounds	Relative Abundance (% of Total Area)		
				AQ	MeOH	EtOAc
1.041	146.1054	$C_6 H_{14} N_2 O_2$	L-Lysine	-	0.02%	-
1.05	132.0896	$C_5 H_{12} N_2 O_2$	Ornithine	0.01%		
1.079	132.0533	$C_4 H_8 N_2 O_3$	Asparagine	0.20%		
1.086	174.1114	$C_6 H_{14} N_4 O_2$	DL-Arginine	1.30%		
1.093	103.0995	$C_5 H_{13} N O$	Choline	1.25%		
1.101	191.07876	$C_7 H_{13} N O_5$	Calystegine C1	0.03%	-	-
1.102	148.0481	$C_4 H_8 N_2 O_4$	Hydroxylated lecithin	0.82%	-	-
1.104	193.03725	$C_9 H_7 N O_4$	5,6-Dihydroxyindole-2-carboxylic acid	-	0.27%	-
1.106	87.03176	$C_3 H_5 N O_2$	2-Iminiopropanoate	0.33%		
1.107	105.0423	$C_3 H_7 N O_3$	L-Serine	0.22%		
1.108	203.0901	$C_7 H_{13} N_3 O_4$	Alanyl asparagine	0.18%		
1.108	133.0372	$C_4 H_7 N O_4$	L-Aspartic acid	0.24%		
1.108	133.03717	$C_4 H_7 N O_4$	4-Hydroxybenzoic acid	0.24%	-	-
1.114	104.01073	$C_3 H_4 O_4$	Malonic acid	0.03%	-	-
1.118	196.05797	$C_6 H_{12} O_7$	Gluconic acid	0.37%	0.54%	-
1.22	117.0789	$C_5 H_{11} N O_2$	L-Valine	-	0.20%	-
1.126	137.0475	$C_7 H_7 N O_2$	Trigonelline	0.54%	0.69%	-
1.128	210.03725	$C_6 H_{10} O_8$	D-Saccharic acid	0.47%	-	-
1.132	210.03728	$C_6 H_{10} O_8$	Galactaric acid	0.01%	0.22%	-
1.132	186.0999	$C_8 H_{14} N_2 O_3$	Alanyl proline	0.08%		
1.133	115.0631	$C_5 H_9 N O_2$	Proline	17.00%		
1.14	162.05261	$C_6 H_{10} O_5$	3-Hydroxymethylglutaric acid	0.46%	1.94%	-
1.164	309.10538	$C_{11} H_{19} N O_9$	N-Acetylneuraminic acid	-	0.90%	-
1.167	90.03157	$C_3 H_6 O_3$	Glyceraldehyde	0.43%	0.74%	-
1.179	322.08954	$C_{12} H_{18} O_{10}$	Lepidimoic acid	-	0.04%	-
1.198	174.05254	$C_7 H_{10} O_5$	Shikimic acid	-	0.41%	0.67%

1.201	116.01078	$C_4 H_4 O_4$	Maleic acid	0.90%	-	0.71%
1.203	138.03151	$C_7 H_6 O_3$	4-Hydroxybenzoic acid	0.07%	-	-
1.205	134.02136	$C_4 H_6 O_5$	L- (-)-Malic acid	2.48%	-	-
1.209	116.01079	$C_4 H_4 O_4$	Fumaric acid	0.01%	1.73%	-
1.223	129.07892	$C_6 H_{11} N O_2$	Pipecolic acid	0.04%	4.26%	-
1.229	516.13246	$C_{18} H_{28} O_{17}$	β-D-Glucuronopyranosyl-(1->3)-a-D-galacturonopyranosyl-(1->2)-L-rhamnose	0.01%	-	-
1.232	85.01624	$C_3 H_3 N O_2$	Cyanoacetic acid	0.23%		
1.236	174.0638	$C_6 H_{10} N_2 O_4$	Formiminoglutamic acid	0.03%		
1.24	146.02125	$C_5 H_6 O_5$	Oxoglutaric acid	0.01%	-	-
1.241	95.03688	$C_5 H_5 N O$	2-Hydroxypyridine	-	0.05%	-
1.245	130.0264	$C_5 H_6 O_4$	Itaconic acid	0.60%	-	-
1.245	174.01587	$C_6 H_6 O_6$	cis-Aconitic acid	-	0.07%	-
1.311	205.0379	$C_{10} H_7 N O_4$	Xanthurenic acid	-	-	0.05%
1.356	106.0264	$C_3 H_6 O_4$	Glyceric acid	-	-	0.10%
1.36	206.04237	$C_7 H_{10} O_7$	2-Methylcitric acid	-	0.02%	0.11%
1.37	129.07886	$C_6 H_{11} N O_2$	Nipecotic acid	-	0.68%	-
1.372	122.0478	$C_6 H_6 N_2 O$	Niacinamide	0.03%	-	-
1.377	156.0059	$C_6 H_4 O_5$	2,5-Furandicarboxylic acid	0.04%		
1.38	130.02647	$C_5 H_6 O_4$	Citraconic acid	0.74%	0.56%	-
1.394	115.0631	$C_5 H_9 N O_2$	L-Proline	T		
1.395	198.0638	$C_8 H_{10} N_2 O_4$	Mimosine	0.06%		
1.414	129.0425	$C_5 H_7 N O_3$	L-Pyroglutamic acid	0.23%		
1.414	119.0584	$C_4 H_9 N O_3$	L-Threonine	0.01%		
1.416	181.0737	$C_9 H_{11} N O_3$	L-Tyrosine		0.08%	
1.419	199.12056	$C_{10} H_{17} N O_3$	Ecgonine methyl ester	0.09%	0.14%	-
1.427	176.03186	$C_6 H_8 O_6$	Ascorbic acid	-	0.24%	0.06%
1.427	176.03184	$C_6 H_8 O_6$	Glucuronic acid-3,6-lactone	0.11%	-	-
1.468	148.03695	$C_5 H_8 O_5$	Citramalic acid	0.01%	-	-
1.472	344.07395	$C_{14} H_{16} O_{10}$	Isovanillic acid glucuronide	0.75%	-	-
1.475	118.02646	$C_4 H_6 O_4$	Succinic acid	0.04%	0.09%	0.12%
1.478	192.06314	$C_7 H_{12} O_6$	D- (-)-Quinic acid	3.13%	0.33%	0.19%
1.479	344.07397	$C_{14} H_{16} O_{10}$	Theogallin	-	3.47%	-

1.521	261.12089	$C_{11} H_{19} N O_6$	Lotaustralin	0.39%	-	-
1.541	192.02679	$C_6 H_8 O_7$	Citric acid	0.18%	-	0.46%
1.545	112.01591	$C_5 H_4 O_3$	2-Furoic acid	0.19%	-	-
1.56	142.0264	$C_6 H_6 O_4$	trans,trans-Muconic acid		0.38%	
1.567	214.1314	$C_{10} H_{18} N_2 O_3$	Valylproline	0.09%		
1.568	131.0944	$C_6 H_{13} N O_2$	6-Aminocaproic acid	0.14%		
1.573	131.0945	$C_6 H_{13} N O_2$	L-Isoleucine		0.19%	
1.608	90.03159	$C_3 H_6 O_3$	L-(+)-Lactic acid	-	-	0.26%
1.609	220.08452	$C_{11} H_{12} N_2 O_3$	5-Hydroxy-L-tryptophan	-	T	-
1.64	185.0687	$C_8 H_{11} N O_4$	2-Aminobicyclohexane-2,6-dicarboxylic acid	-	0.01%	-
1.658	240.0741	$C_{10} H_{12} N_2 O_5$	(±)-2-(1-Methylpropyl)-4,6-dinitrophenol	-	0.01%	-
1.668	161.0687	$C_6 H_{11} N O_4$	Aminoadipic acid	-	0.11%	-
1.668	294.0374	$C_{13} H_{10} O_8$	Banksiamarin B	-	-	0.15%
1.679	85.08901	$C_5 H_{11} N$	Piperidine	-	-	0.16%
1.701	92.02946	$C_3 H_8 O S$	Cibulins	-	-	0.58%
1.737	174.01629	$C_6 H_6 O_6$	trans-Aconitic acid	0.24%	0.03%	-
1.737	136.03701	$C_4 H_8 O_5$	Erythronic acid	T	-	-
1.739	130.02642	$C_5 H_6 O_4$	Glutaconic acid	0.04%	-	-
1.747	98.03663	$C_5 H_6 O_2$	2-Furanmethanol	-	-	0.09%
1.749	206.04246	$C_7 H_{10} O_7$	3-Hydroxy-3-(methoxycarbonyl) pentane dioic acid	-	0.05%	-
1.768	332.07417	$C_{13} H_{16} O_{10}$	6-Galloylglucose	6.06%	3.97%	3.39%
1.778	312.04795	$C_{13} H_{12} O_9$	Caftaric acid	0.02%	-	-
1.785	342.11611	$C_{12} H_{22} O_{11}$	α, α-Trehalose	6.86%	12.27%	23.22%
1.793	244.0581	$C_{10} H_{12} O_7$	1-O-Galloylglycerol	-	-	0.02%
1.813	88.0159	$C_3 H_4 O_3$	Pyruvic acid	0.24%	-	-
1.824	147.05303	$C_5 H_9 N O_4$	L-Glutamic acid	-	0.19%	-
1.858	144.04211	$C_6 H_8 O_4$	5-hydroxy-4-methoxy-5,6-dihydro-2H-pyran-2-one	0.89%	0.17%	-
1.923	316.07932	$C_{13} H_{16} O_9$	Ginnalin B	0.01%	-	-
1.976	88.05237	$C_4 H_8 O_2$	Butyric acid	-	-	0.01%
2.027	134.02145	$C_4 H_6 O_5$	DL-Malic acid	0.05%	0.07%	-
2.037	153.0788	$C_8 H_{11} N O_2$	3,5-Dimethoxyaniline	-	-	0.13%

2.193	288.08452	$C_{12}H_{16}O_8$	Phlorin	-	0.02%	-
2.142	108.0211	$C_6H_4O_2$	1,2-Benzoquinone	0.04%		
2.2	219.11062	$C_9H_{17}NO_5$	Pantothenic acid	-	0.02%	-
2.214	134.0215	$C_4H_6O_5$	Malic acid	-	-	0.05%
2.244	166.06293	$C_9H_{10}O_3$	Apocynin	0.02%	0.04%	-
2.397	154.02654	$C_7H_6O_4$	Gentisic acid	0.02%	0.05%	-
2.421	328.1157	$C_{15}H_{20}O_8$	Ethylvanillin glucoside	-	-	0.01%
2.433	124.0272	$C_5H_4N_2O_2$	Pyrazin-2-carboxylic acid	-	0.01%	-
2.645	356.03816	$C_{14}H_{12}O_{11}$	(+)-Chebulic acid	0.54%	-	-
2.768	182.069	$C_8H_{10}N_2O_3$	3-Hydroxy-5-(hydroxymethyl)-2-methylisonicotinaldehyde oxime	-	0.14%	-
2.844	212.0684	$C_{10}H_{12}O_5$	Vanillactic acid	T		
2.911	302.06383	$C_{12}H_{14}O_9$	Pyrogallol-2-O-glucuronide	-	0.21%	-
2.938	158.0579	$C_7H_{10}O_4$	Succinylacetone		T	
3.059	204.08978	$C_{11}H_{12}N_2O_2$	L-Tryptophan	-	0.03%	-
3.06	187.06318	$C_{11}H_9NO_2$	trans-3-Indoleacrylic acid	0.11%	0.32%	-
3.061	145.05266	C_9H_7NO	4-Indolecarbaldehyde	-	0.04%	-
3.094	390.11625	$C_{16}H_{22}O_{11}$	2,3,4,5,6-Penta-O-acetyl-D-glucose	0.01%	0.01%	-
3.11	484.08475	$C_{20}H_{20}O_{14}$	Gallic acid 3-O-(6-galloylglucoside)	0.74%	-	-
3.145	228.1472	$C_{11}H_{20}N_2O_3$	Prolylleucine	0.08%		
3.485	496.08487	$C_{21}H_{20}O_{14}$	(1S,3R,4R,5R)-1,3-dihydroxy-4,5-bis(3,4,5-trihydroxybenzoyloxy) cyclohexane-1-carboxylic acid	-	0.28%	-
3.569	218.14169	$C_{13}H_{18}N_2O$	5-Methoxydimethyltryptamine	0.01%	-	-
3.652	112.05232	$C_6H_8O_2$	(2E,4E)-2,4-Hexadienoic acid	0.08%	-	-
3.797	314.10014	$C_{14}H_{18}O_8$	Vanilloside	-	0.01%	-
3.94	185.1049	$C_9H_{15}NO_3$	Pseudoecgonine	-	-	0.02%
4.647	96.021	$C_5H_4O_2$	2-Furancarboxaldehyde	-	0.02%	-
4.659	154.02638	$C_7H_6O_4$	2,4-Dihydroxybenzoic acid	-	0.15%	-
4.667	122.03663	$C_7H_6O_2$	4-Hydroxybenzaldehyde	-	0.49%	0.10%
4.756	342.0951	$C_{15}H_{18}O_9$	Glucocaffeic acid	-	-	0.01%
4.921	292.02154	$C_{13}H_8O_8$	Brevifolincarboxylic acid	0.09%	-	-
4.93	360.10563	$C_{15}H_{20}O_{10}$	Glucosyringic acid	-	0.07%	-

5.164	176.06843	$C_7 H_{12} O_5$	2-Isopropylmalic acid	0.01%	0.02%	0.03%
5.757	216.08974	$C_{12} H_{12} N_2 O_2$	L-1,2,3,4-Tetrahydro-beta-carboline-3-carboxylic acid	-	0.01%	-
6.475	296.0529	$C_{13} H_{12} O_8$	Caffeoylmalic acid	0.05%	-	-
6.812	187.06319	$C_{11} H_9 N O_2$	Indoleacrylic acid	T	-	-
6.827	230.10541	$C_{13} H_{14} N_2 O_2$	(1xi,3xi)-1,2,3,4-Tetrahydro-1-methyl-beta-carboline-3-carboxylic acid	-	0.02%	-
6.939	329.1988	$C_{20} H_{27} N O_3$	Trilostane		0.03%	
8.333	636.09597	$C_{27} H_{24} O_{18}$	1,3,6-Tri-O-galloyl-beta-D-glucose	-	0.25%	0.93%
8.44	484.08546	$C_{20} H_{20} O_{14}$	1,6-Bis-O-(3,4,5-trihydroxybenzoyl) hexopyranose	5.13%	5.19%	2.52%
8.726	634.08076	$C_{27} H_{22} O_{18}$	Sanguiin H4	0.84%	-	0.07%
8.941	512.15318	$C_{23} H_{28} O_{13}$	3-(4-Hydroxy-3-methoxyphenyl)-1,2-propanediol 2-O-(galloyl-glucoside)	T	-	-
8.945	478.07477	$C_{21} H_{18} O_{13}$	Miquelianin	0.04%	0.42%	-
8.948	308.0528	$C_{14} H_{12} O_8$	Fulvic acid	0.02%		
8.985	478.07427	$C_{21} H_{18} O_{13}$	Quercetin 3-O-glucuronide	-	0.08%	-
9.105	470.01185	$C_{21} H_{10} O_{13}$	Sanguisorbic acid dilactone	0.56%	0.88%	-
9.21	126.03151	$C_6 H_6 O_3$	Phloroglucinol	0.10%	1.93%	-
9.291	498.1009	$C_{21} H_{22} O_{14}$	Methyl 4,6-di-O-galloyl-beta-D-glucopyranoside	T	-	-
9.532	338.09996	$C_{16} H_{18} O_8$	3-O-p-Coumaroylquinic acid	T	-	-
9.538	314.06351	$C_{13} H_{14} O_9$	Beta-D-Glucopyranuronic acid	2.37%	0.58%	0.33%
9.738	126.03149	$C_6 H_6 O_3$	Maltol	0.02%	0.06%	-
9.74	236.06837	$C_{12} H_{12} O_5$	Dillapional	0.01%	-	-
9.817	374.12117	$C_{16} H_{22} O_{10}$	Geniposidic acid	-	0.02%	-
9.89	402.15262	$C_{18} H_{26} O_{10}$	Benzyl beta-primeveroside	T	0.03%	-
10.064	636.09617	$C_{27} H_{24} O_{18}$	1,2,6-Trigalloyl-β-D-glucopyranose	0.09%	5.44%	2.85%
10.235	96.02095	$C_5 H_4 O_2$	2H-Pyran-2-one	0.05%	0.09%	1.02%
10.237	126.03161	$C_6 H_6 O_3$	Pyrogallol	6.28%	5.28%	2.19%
10.357	524.15327	$C_{24} H_{28} O_{13}$	Barbatoflavan	0.01%	-	-
10.382	160.05219	$C_{10} H_8 O_2$	6-Methylcoumarin	-	0.01%	-
10.406	126.0316	$C_6 H_6 O_3$	1,2,3-Trihydroxybenzene	-	-	0.15%
10.448	432.12706	$C_{18} H_{24} O_{12}$	Apiosylglucosyl 4-hydroxybenzoate	T	-	-

10.475	484.08519	$C_{20}H_{20}O_{14}$	Hamamelitannin	2.10%	0.02%	0.62%
10.484	296.05287	$C_{13}H_{12}O_8$	cis-Coutaric acid	0.33%	0.86%	0.19%
10.577	478.07496	$C_{21}H_{18}O_{13}$	6-Hydroxyluteolin 6-glucuronide	0.01%	-	-
10.691	196.03709	$C_9H_8O_5$	3-Methoxy-4,5-methylenedioxybenzoic acid	T	-	-
10.703	372.10552	$C_{16}H_{20}O_{10}$	Veranisatin C	0.02%	0.04%	-
10.706	198.05269	$C_9H_{10}O_5$	Syringic acid	0.16%	0.15%	-
10.796	448.10018	$C_{21}H_{20}O_{11}$	(1ξ)-1,5-Anhydro-1-[2-(3,4-dihydroxyphenyl)-5,7-dihydroxy-4-oxo-4H-chromen-8-yl]-D-galactitol	-	0.03%	-
10.879	584.13777	$C_{25}H_{28}O_{16}$	Neomangiferin	-	T	-
11.055	466.11077	$C_{21}H_{22}O_{12}$	(2R,3R)-2-(3,4-dihydroxyphenyl)-5,7-dihydroxy-3-{[(2S,3R,4S,5S,6R)-3,4,5-trihydroxy-6-(hydroxymethyl) oxan-2-yl] oxy}-3,4-dihydro-2H-1-benzopyran-4-one	0.01%	-	-
11.057	302.00606	$C_{14}H_6O_8$	Ellagic acid	2.45%	10.74%	33.49%
11.159	236.03157	$C_{11}H_8O_6$	6-Methoxy-8-hydroxyisocoumarin-3-carboxylic acid	-	0.58%	-
11.257	304.05785	$C_{15}H_{12}O_7$	Nigrescin	T	0.02%	-
11.263	174.08907	$C_8H_{14}O_4$	Suberic acid	T	-	0.06%
11.272	610.15272	$C_{27}H_{30}O_{16}$	Quercetin 3-O-rhamnoside-7-O-glucoside	0.05%	-	-
11.295	610.15295	$C_{27}H_{30}O_{16}$	Rutin	-	0.14%	0.10%
11.3	464.09518	$C_{21}H_{20}O_{12}$	3,5,7-trihydroxy-2-(3-hydroxy-4-{[(2S,3R,4S,5S,6R)-3,4,5-trihydroxy-6-(hydroxymethyl) oxan-2-yl]oxy}phenyl)-4H-chromen-4-one	-	0.02%	-
11.341	436.10043	$C_{20}H_{20}O_{11}$	Taxifolin 3-apioside	T	-	-
11.348	184.07305	$C_9H_{12}O_4$	Vanylglycol	0.01%	-	-
11.372	498.13718	$C_{22}H_{26}O_{13}$	Verproside	0.03%	0.07%	-
11.473	464.09505	$C_{21}H_{20}O_{12}$	Myricitrin	0.05%	0.13%	0.19%
11.487	168.126	$C_9H_{16}N_2O$	N-Methylloline	-	-	0.01%
11.507	554.16359	$C_{25}H_{30}O_{14}$	Lippioside II	T	-	-
11.527	388.13676	$C_{17}H_{24}O_{10}$	Geniposide	0.01%	0.01%	-
11.529	422.08464	$C_{19}H_{18}O_{11}$	1,5,8-Trihydroxy-9-oxo-9H-xanthen-3-yl beta-D-glucopyranoside	0.02%	0.05%	-

11.533	400.1519	$C_{22} H_{24} O_7$	Melafolone	T	-	-
11.546	522.21046	$C_{26} H_{34} O_{11}$	Isolariciresinol 9-O-beta-D-glucoside	0.01%	-	-
11.55	184.10991	$C_{10} H_{16} O_3$	(1'R)-Nepetalic acid	T	-	-
11.553	492.09059	$C_{22} H_{20} O_{13}$	Carminic acid	0.04%	-	-
11.586	164.05849	$C_8 H_8 N_2 O_2$	Ricinine	0.01%	0.03%	0.12%
11.666	216.09953	$C_{10} H_{16} O_5$	(4S,5S,8S,10R)-4,5,8-trihydroxy-10-methyl-3,4,5,8,9,10-hexahydro-2H-oxecin-2-one	0.05%	-	-
11.674	478.11101	$C_{22} H_{22} O_{12}$	6-Methoxyluteolin 7-glucoside	0.01%	-	-
11.692	132.04297	$C_6 H_4 N_4$	Pteridine	-	0.01%	-
11.694	164.04717	$C_9 H_8 O_3$	2-Hydroxycinnamic acid	0.02%	0.05%	-
11.788	260.0316	$C_{13} H_8 O_6$	Urolithin D	-	0.02%	-
11.953	448.10033	$C_{21} H_{20} O_{11}$	Trifolin	0.01%	-	-
12.018	184.03695	$C_8 H_8 O_5$	Methyl gallate	0.25%	11.39%	-
12.019	170.02144	$C_7 H_6 O_5$	Gallic acid	26.23%	8.64%	2.60%
12.048	302.04238	$C_{15} H_{10} O_7$	Quercetin	0.11%	0.33%	0.17%
12.053	448.1	$C_{21} H_{20} O_{11}$	5-(5,7-Dihydroxy-3-methoxy-4-oxo-4H-chromen-2-yl)-2-hydroxyphenyl beta-D-xylopyranoside	-	0.05%	-
12.06	150.06809	$C_9 H_{10} O_2$	Hydrocinnamic acid	T	-	0.14%
12.138	396.15682	$C_{23} H_{24} O_6$	Glabrachalcone	T	-	-
12.155	448.06386	$C_{20} H_{16} O_{12}$	Ellagic acid 2-rhamnoside	0.11%	1.44%	0.69%
12.183	448.09955	$C_{21} H_{20} O_{11}$	Maritimein	-	0.01%	0.02%
12.184	456.10534	$C_{23} H_{20} O_{10}$	Epicatechin 3-O- (3-O-methylgallate)	-	0.02%	-
12.38	334.03252	$C_{15} H_{10} O_9$	3,5,6,7,2',3',4'-Heptahydroxyflavone	-	0.20%	-
12.459	462.0795	$C_{21} H_{18} O_{12}$	Aureusidin 6-glucuronide	-	-	2.52%
12.509	610.1894	$C_{28} H_{34} O_{15}$	Neohesperidin	-	-	0.16%
12.539	292.1209	$C_{18} H_{16} N_2 O_2$	4-phenyl-2-[(3-pyridylamino) methylidene] cyclohexane-1,3-dione	-	0.02%	-
12.584	498.17406	$C_{23} H_{30} O_{12}$	Eucaglobulin	-	0.02%	-
12.711	262.04766	$C_{13} H_{10} O_6$	Maclurin	-	0.02%	-
12.82	188.1047	$C_9 H_{16} O_4$	Azelaic acid	-	-	0.33%
12.822	362.17305	$C_{20} H_{26} O_6$	Secoisolariciresinol	-	T	-
12.843	580.21589	$C_{28} H_{36} O_{13}$	(+)-7-epi-Syringaresinol 4'-glucoside	-	0.01%	-

12.844	518.1785	$C_{26} H_{30} O_{11}$	Phellodensin E	-	-	0.05%
12.924	302.11519	$C_{17} H_{18} O_5$	Lusianin	0.01%	-	-
13.088	241.1675	$C_{13} H_{23} N O_3$	Dioscoretine	-	-	0.05%
13.307	556.15817	$C_{28} H_{28} O_{12}$	Epicatechin 5-O-beta-D-glucopyranoside-3-benzoate	T	-	-
13.38	416.1681	$C_{19} H_{28} O_{10}$	Phenylethyl primeveroside	T	-	-
13.41	170.02148	$C_7 H_6 O_5$	Phloroglucinic acid	0.98%	1.80%	4.33%
13.428	442.1992	$C_{25} H_{30} O_7$	Exiguaflavanone M	-	-	0.13%
13.455	462.11665	$C_{22} H_{22} O_{11}$	Leptosin	-	0.01%	-
13.51	169.11009	$C_9 H_{15} N O_2$	Homoarecoline	T	-	-
13.527	191.0944	$C_{11} H_{13} N O_2$	5-Methoxytryptophol	-	-	0.06%
13.593	502.1836	$C_{26} H_{30} O_{10}$	Flavaprin	-	-	0.07%
13.611	358.14156	$C_{20} H_{22} O_6$	Pedicellin	0.01%	-	-
13.701	308.07953	$C_{17} H_{12} N_2 O_4$	Flazine	-	0.01%	-
13.725	330.03743	$C_{16} H_{10} O_8$	Blighinone	0.12%	-	-
13.822	227.09433	$C_{14} H_{13} N O_2$	Koenoline	T	T	-
13.898	326.115	$C_{19} H_{18} O_5$	4',7-Dimethoxy-6,8-dimethyl-5-hydroxyflavone	T	-	T
13.928	554.2148	$C_{30} H_{34} O_{10}$	Lappaol C	-	T	-
13.958	520.1948	$C_{26} H_{32} O_{11}$	Matairesinoside	-	-	0.01%
14.024	500.1674	$C_{26} H_{28} O_{10}$	Ikarisoside A	-	-	0.03%
14.045	488.20476	$C_{26} H_{32} O_9$	Terretonin	T	-	-
14.277	444.17847	$C_{24} H_{28} O_8$	Astellolide F	-	0.02%	-
14.318	178.06285	$C_{10} H_{10} O_3$	4-Methoxycinnamic acid	-	0.01%	-
14.364	312.13574	$C_{19} H_{20} O_4$	Desmosdumotin C	T	-	0.02%
14.472	376.1882	$C_{22} H_{24} N_4 O_2$	N-{(3S,5S)-1-Methyl-5-[3-(4-methylphenyl)-1,2,4-oxadiazol-5-yl]-3-pyrrolidinyl}-2-phenylacetamide	-	0.05%	-
14.475	208.10971	$C_{12} H_{16} O_3$	β-Asarone	-	0.04%	-
14.561	226.1203	$C_{12} H_{18} O_4$	Allixin	-	-	0.03%
14.742	584.22534	$C_{31} H_{36} O_{11}$	hemsleyanoside	-	0.02%	-
15.119	456.1776	$C_{25} H_{28} O_8$	Lobaric acid	-	-	0.02%
15.874	328.2249	$C_{18} H_{32} O_5$	Corchorifatty acid F	T	0.01%	0.29%
15.912	452.18324	$C_{26} H_{28} O_7$	Derrichalcone	T	-	-

16.002	444.2512	$C_{26} H_{36} O_6$	Pyrenulic acid C	-	-	0.19%
16.09	212.0947	$C_{13} H_{12} N_2 O$	Harmine	-	-	0.08%
16.301	470.1941	$C_{26} H_{30} O_8$	Limonin	-	-	0.14%
16.382	212.1407	$C_{12} H_{20} O_3$	Cucurbic acid	-	-	0.01%
16.478	540.1663	$C_{25} H_{32} O_{11} S$	Sumalarin B	-	-	0.02%
16.51	256.07345	$C_{15} H_{12} O_4$	Isoliquiritigenin	-	T	0.01%
16.532	316.1308	$C_{18} H_{20} O_5$	Methylodoratol	-	-	0.02%
16.697	498.1884	$C_{27} H_{30} O_9$	Solanoeclepin A	-	-	0.13%
16.787	472.2097	$C_{26} H_{32} O_8$	Kushenol H	-	-	0.07%
16.844	344.0532	$C_{17} H_{12} O_8$	3,4,3'-Tri-O-methylellagic acid	-	-	0.01%
16.889	300.1361	$C_{18} H_{20} O_4$	Angoletin	-	-	T
16.968	286.08397	$C_{16} H_{14} O_5$	Homobutein	-	T	0.01%
17.064	440.1828	$C_{25} H_{28} O_7$	Lonchocarpol E	-	-	0.15%
17.209	544.2668	$C_{30} H_{40} O_9$	Physagulin F	-	-	0.10%
17.217	149.0839	$C_9 H_{11} N O$	Venoterpine	-	-	0.05%
17.219	184.0997	$C_{12} H_{12} N_2$	Harmalan	-	-	0.11%
17.237	822.40293	$C_{42} H_{62} O_{16}$	Glycyrrhizin	T	-	-
17.253	281.1408	$C_{18} H_{19} N O_2$	Floribundine	-	-	0.01%
17.316	180.11485	$C_{11} H_{16} O_2$	Jasmolone	-	0.61%	1.48%
17.341	310.12026	$C_{19} H_{18} O_4$	Castillene B	T	-	-
17.368	282.12542	$C_{18} H_{18} O_3$	Ohobanin	0.01%	T	0.09%
17.481	136.05228	$C_8 H_8 O_2$	4-Methoxybenzaldehyde	-	0.04%	0.83%
17.615	188.0835	$C_{12} H_{12} O_2$	Trigoforin	-	-	0.11%
17.633	286.2141	$C_{16} H_{30} O_4$	Hexadecanedioic acid	-	-	0.04%
17.709	540.199	$C_{29} H_{32} O_{10}$	Pilosanol A	-	-	0.03%
17.755	284.14097	$C_{18} H_{20} O_3$	Minquartynoic acid	T	-	-
17.763	342.1465	$C_{20} H_{22} O_5$	Brosimacutin C	-	-	0.03%
17.878	408.2296	$C_{26} H_{32} O_4$	Methyllinderatin	-	-	0.05%
17.902	424.1881	$C_{25} H_{28} O_6$	Paratocarpin G	T	-	-
17.949	456.21436	$C_{26} H_{32} O_7$	Antiarone J	-	0.54%	-
18.124	372.2509	$C_{20} H_{36} O_6$	Sterebin Q4	-	-	0.01%
18.164	328.1309	$C_{19} H_{20} O_5$	2',3',4',6'-Tetramethoxychalcone	-	-	0.05%

18.237	428.1831	$C_{24}H_{28}O_7$	Heteroflavanone B	-	-	0.05%
18.392	292.2035	$C_{18}H_{28}O_3$	9S,13R-12-Oxophytodienoic acid	-	-	0.15%
18.409	296.1411	$C_{19}H_{20}O_3$	(1E)-1,7-bis(4-hydroxyphenyl) hept-1-en-3-one	-	0.60%	-
18.442	342.11601	$C_{12}H_{22}O_{11}$	D-(+)-Maltose	-	0.51%	-
18.444	141.1152	$C_8H_{15}NO$	(R)-Pelletierine	-	-	0.03%
18.47	466.19888	$C_{27}H_{30}O_7$	Eriotriochin	-	0.50%	-
18.513	288.2299	$C_{16}H_{32}O_4$	(S)-10,16-Dihydroxyhexadecanoic acid	-	-	0.03%
18.542	296.141	$C_{19}H_{20}O_3$	(1E)-1,7-bis(4-hydroxyphenyl) hept-1-en-3-one	-	-	0.56%
18.605	414.2037	$C_{24}H_{30}O_6$	Bis(4-ethylbenzylidene) sorbitol	-	-	1.96%
18.712	268.1462	$C_{18}H_{20}O_2$	Diethylstilbesterol	-	-	0.07%
18.72	268.13078	$C_{14}H_{20}O_5$	Kamahine C	0.02%	0.01%	-
18.735	278.22429	$C_{18}H_{30}O_2$	alpha-Linolenic acid	-	0.51%	0.22%
18.748	208.10964	$C_{12}H_{16}O_3$	Isoelemicin	0.01%	-	0.19%
18.767	526.25646	$C_{30}H_{38}O_8$	Kosamol A	-	0.52%	-
18.814	144.1147	$C_8H_{16}O_2$	Caprylic acid	-	-	0.01%
18.824	300.0997	$C_{17}H_{16}O_5$	2',4'-Dihydroxy-3,4-dimethoxychalcone	-	-	0.07%
18.863	134.073	$C_9H_{10}O$	2,4-Dimethylbenzaldehyde	-	-	0.63%
18.865	118.0782	C_9H_{10}	beta-Methylstyrene	-	-	1.98%
18.899	554.28739	$C_{32}H_{42}O_8$	11β-hydroxycolossolactone VIII	0.01%	-	-
18.987	372.1208	$C_{20}H_{20}O_7$	Tangeretin	-	-	0.03%
19.444	132.0573	C_9H_8O	Cinnamaldehyde	-	-	0.09%
19.605	226.09933	$C_{15}H_{14}O_2$	7-Hydroxyflavan	-	0.84%	-
19.657	304.1669	$C_{18}H_{24}O_4$	2-Hydroxyestriol	-	-	1.12%
19.715	272.1406	$C_{17}H_{20}O_3$	(S)-Verimol F	-	-	0.75%
21.005	592.2693	$C_{35}H_{36}N_4O_5$	Pheophorbide a	-	-	0.15%
21.418	356.32872	$C_{22}H_{44}O_3$	2(R)-hydroxydocosanoic acid	T	-	0.04%
22.345	472.3551	$C_{30}H_{48}O_4$	Maslinic acid	-	-	0.64%
**Grand Total**				**100%**	**100%**	**100%**

Table S2: LC-MS putative identification and % relative abundance of phytochemicals identified in the extracts of *Terminalia chebula*. Compounds less than 0.01% of the total area were considered as trace amounts and denoted as T.

Retention time [min]	Molecular weight	Empirical Formula	Putative Compounds	Relative Abundance (% of Total Area)		
				**AQ**	**MeOH**	**EtOAc**
1.205	114.04278	$C_4 H_6 N_2 O_2$	L-3-Cyanoalanine	-	0.04%	-
1.22	87.03192	$C_3 H_5 N O_2$	2-Aminoacrylic acid	-	0.15%	-
1.221	189.09992	$C_8 H_{15} N O_4$	N-Methylcalystegine B2	-	0.18%	-
1.222	276.09543	$C_{10} H_{16} N_2 O_7$	gamma-Glutamylglutamic acid	-	1.08%	-
1.223	342.11587	$C_{12} H_{22} O_{11}$	α,α-Trehalose	-	3.43%	-
1.224	105.0424	$C_3 H_7 N O_3$	L-Serine	-	0.03%	-
1.225	215.0791	$C_9 H_{13} N O_5$	N-Methacryloyl-L-glutamic acid	-	0.61%	-
1.244	132.04206	$C_5 H_8 O_4$	Methylsuccinic acid	-	11.54%	-
1.246	210.03738	$C_6 H_{10} O_8$	Galactaric acid	-	0.31%	-
1.279	356.13167	$C_{13} H_{24} O_{11}$	4-O-Methylgalactinol	-	0.07%	-
1.296	144.04208	$C_6 H_8 O_4$	Methylglutamic acid	-	0.29%	-
1.298	196.05798	$C_6 H_{12} O_7$	Galactonic acid	-	-	0.10%
1.3	145.08502	$C_5 H_{11} N_3 O_2$	4-Guanidinobutyric acid	-	0.22%	-
1.376	342.11598	$C_{12} H_{22} O_{11}$	Melibiose	-	0.05%	-
1.357	160.03685	$C_6 H_8 O_5$	Oxoadipic acid	-	-	0.34%
1.383	134.02136	$C_4 H_6 O_5$	L-(-)-Malic acid	-	-	1.09%
1.428	129.07882	$C_6 H_{11} N O_2$	Pipecolic acid	0.02%	0.01%	0.03%
1.444	174.11148	$C_6 H_{14} N_4 O_2$	DL-Arginine	1.45%	-	-
1.479	132.05333	$C_4 H_8 N_2 O_3$	Asparagine	0.31%	0.23%	-
1.479	187.1207	$C_9 H_{17} N O_3$	(S)-(+)-N-Boc-3-pyrrolidinol	-	0.21%	-
1.481	103.09955	$C_5 H_{13} N O$	Choline	2.23%	1.61%	-
1.484	182.07877	$C_6 H_{14} O_6$	L-Iditol	3.34%	7.32%	7.23%
1.486	90.03157	$C_3 H_6 O_3$	Glyceraldehyde	5.35%	11.03%	26.76%
1.489	86.03662	$C_4 H_6 O_2$	gamma-Butyrolactone	0.28%	0.11%	-
1.492	294.10599	$C_{10} H_{18} N_2 O_8$	Distichonic acid A	0.37%	-	-
1.492	133.03731	$C_4 H_7 N O_4$	L-Aspartic acid	0.32%	0.16%	0.03%

1.495	147.05287	$C_5 H_9 N O_4$	L-Glutamic acid	1.67%	1.15%	-
1.496	192.06305	$C_7 H_{12} O_6$	Quinic acid	0.39%	-	1.53%
1.501	196.05798	$C_6 H_{12} O_7$	Gluconic acid	0.39%	0.18%	-
1.501	117.07878	$C_5 H_{11} N O_2$	L-Valine	1.09%	0.90%	-
1.501	129.04249	$C_5 H_7 N O_3$	L-Pyroglutamic acid	-	0.46%	-
1.505	137.04746	$C_7 H_7 N O_2$	Trigonelline	0.24%	0.19%	-
1.506	210.03733	$C_6 H_{10} O_8$	D-Saccharic acid	1.03%	-	-
1.511	144.04206	$C_6 H_8 O_4$	5-hydroxy-4-methoxy-5,6-dihydro-2H-pyran-2-one	0.59%	0.64%	-
1.526	104.01072	$C_3 H_4 O_4$	Malonic acid	0.02%	-	-
1.548	180.06314	$C_6 H_{12} O_6$	D-(+)-Glucose	0.88%	-	-
1.554	148.03698	$C_5 H_8 O_5$	D-Xylono-1,5-lactone	0.28%	-	-
1.562	118.02649	$C_4 H_6 O_4$	Succinic acid	-	0.04%	0.08%
1.566	90.03152	$C_3 H_6 O_3$	L-(+)-Lactic acid	-	-	0.34%
1.568	129.04239	$C_5 H_7 N O_3$	4-Oxoproline	-	-	0.44%
1.572	138.03148	$C_7 H_6 O_3$	4-Hydroxybenzoic acid	0.23%	0.25%	-
1.572	94.04171	$C_6 H_6 O$	Phenol	0.56%	-	-
1.573	344.07409	$C_{14} H_{16} O_{10}$	Theogallin	-	0.03%	0.02%
1.58	116.01077	$C_4 H_4 O_4$	Maleic acid	0.68%	-	0.39%
1.58	134.02133	$C_4 H_6 O_5$	D-(+)-Malic acid	1.77%	1.65%	-
1.601	185.10486	$C_9 H_{15} N O_3$	Pseudoecgonine	0.07%	0.02%	0.01%
1.607	155.09432	$C_8 H_{13} N O_2$	Arecoline	0.05%	-	-
1.632	115.06327	$C_5 H_9 N O_2$	L-Proline	-	0.26%	-
1.654	214.13153	$C_{10} H_{18} N_2 O_3$	Valylproline	-	0.15%	0.22%
1.702	109.05256	$C_6 H_7 N O$	Nicotinyl alcohol	0.31%	0.25%	-
1.745	192.0267	$C_6 H_8 O_7$	Citric acid	0.97%	-	0.21%
1.748	112.01588	$C_5 H_4 O_3$	2-Furoic acid	0.64%	0.52%	-
1.748	88.0159	$C_3 H_4 O_3$	Pyruvic acid	0.15%	0.05%	0.01%
1.751	174.01619	$C_6 H_6 O_6$	cis-Aconitic acid	0.33%	-	-
1.773	90.03153	$C_3 H_6 O_3$	L-Lactic acid	0.09%	0.49%	-
1.775	181.07364	$C_9 H_{11} N O_3$	L-Tyrosine	0.54%	0.45%	-
1.778	96.02097	$C_5 H_4 O_2$	2-Furancarboxaldehyde	0.18%	0.20%	0.02%
1.778	129.0424	$C_5 H_7 N O_3$	D-(+)-Pyroglutamic Acid	0.47%	-	-

1.784	302.06328	$C_{12} H_{14} O_9$	Pyrogallol-2-O-glucuronide	0.07%	-	-
1.785	199.12043	$C_{10} H_{17} N O_3$	Ecgonine methyl ester	0.14%	0.17%	-
1.809	206.04257	$C_7 H_{10} O_7$	2-Methylcitric acid	-	0.03%	-
1.82	354.15268	$C_{14} H_{26} O_{10}$	Isopropyl apiosylglucoside	-	0.07%	-
1.847	202.02667	$C_{11} H_6 O_4$	Bergaptol	0.02%	T	-
1.849	131.09458	$C_6 H_{13} N O_2$	L-Isoleucine	0.12%	0.09%	-
1.887	224.06841	$C_{11} H_{12} O_5$	Elenolide	-	0.01%	-
1.905	236.0684	$C_{12} H_{12} O_5$	Dillapional	-	T	0.01%
1.912	98.03661	$C_5 H_6 O_2$	5-Hydroxy-4-pentenoic acid d-lactone	0.23%	-	-
1.93	85.08905	$C_5 H_{11} N$	Piperidine	0.34%	-	0.10%
1.975	149.08397	$C_9 H_{11} N O$	Venoterpine	0.04%	-	-
2.01	210.05269	$C_{10} H_{10} O_5$	5-Hydroxyferulic acid	-	0.07%	-
2.022	254.07863	$C_6 H_{15} N_4 O_5 P$	L-Phosphoarginine	-	0.01%	-
2.045	294.03749	$C_{13} H_{10} O_8$	Banksiamarin B	0.17%	0.05%	0.13%
2.053	100.05235	$C_5 H_8 O_2$	Senecioic acid	0.30%	-	-
2.058	174.05268	$C_7 H_{10} O_5$	Shikimic acid	2.64%	3.14%	4.80%
2.13	256.05811	$C_{11} H_{12} O_7$	(2R,3S)-Piscidic acid	0.01%	-	-
2.169	115.06323	$C_5 H_9 N O_2$	Proline	0.28%	-	-
2.175	222.05248	$C_{11} H_{10} O_5$	Isofraxidin	0.02%	0.04%	-
2.176	178.06283	$C_{10} H_{10} O_3$	4-Methoxycinnamic acid	0.01%	0.05%	0.01%
2.18	259.08409	$C_{14} H_{13} N O_4$	Skimmianine	0.02%	-	-
2.184	236.03185	$C_{11} H_8 O_6$	Methylspinazarin	-	0.08%	-
2.237	154.02646	$C_7 H_6 O_4$	Gentisic acid	0.01%	-	0.09%
2.277	165.07876	$C_9 H_{11} N O_2$	L-Phenylalanine	0.12%	0.11%	0.10%
2.284	190.02635	$C_{10} H_6 O_4$	8H-1,3-Dioxolo[4,5-h][1] benzopyran-8-one	0.26%	-	-
2.285	290.09975	$C_{12} H_{18} O_8$	Osmundalin	0.04%	0.03%	-
2.296	448.15769	$C_{19} H_{28} O_{12}$	8-O-Acetyl shanzhiside methyl ester	0.19%	0.18%	-
2.3	248.06824	$C_{13} H_{12} O_5$	Coriandrone E	0.01%	0.01%	0.01%
2.356	332.07411	$C_{13} H_{16} O_{10}$	6-Galloylglucose	3.74%	0.68%	-
2.38	134.02141	$C_4 H_6 O_5$	Malic acid	0.08%	-	-
2.444	448.06386	$C_{20} H_{16} O_{12}$	Ellagic acid 2-rhamnoside	0.01%	0.21%	0.33%
2.496	128.04739	$C_6 H_8 O_3$	Osmundalactone	-	0.01%	-

2.51	292.02177	$C_{13} H_8 O_8$	Brevifolincarboxylic acid	0.11%	0.03%	0.61%
2.523	374.12161	$C_{16} H_{22} O_{10}$	Geniposidic acid	T	-	-
2.527	279.11064	$C_{14} H_{17} N O_5$	Niazirin	T	0.07%	-
2.56	288.0844	$C_{12} H_{16} O_8$	Phlorin	0.03%	0.02%	-
2.618	234.01615	$C_{12} H_{10} O S_2$	Arctinol A	T	-	-
2.718	110.03671	$C_6 H_6 O_2$	Catechol	0.10%	0.11%	0.15%
2.72	154.02652	$C_7 H_6 O_4$	Protocatechuic acid	0.07%	-	-
2.804	370.05335	$C_{15} H_{14} O_{11}$	2-O-Caffeoylhydroxycitric acid	-	0.43%	-
2.849	314.06336	$C_{13} H_{14} O_9$	Beta-D-Glucopyranuronic acid	0.18%	0.55%	0.49%
2.937	272.05311	$C_{11} H_{12} O_8$	Fukiic acid	0.02%	0.94%	0.77%
3.158	212.06824	$C_{10} H_{12} O_5$	Propyl gallate	0.30%	0.35%	0.12%
3.183	140.04729	$C_7 H_8 O_3$	Ethyl maltol	T	-	-
3.432	228.14714	$C_{11} H_{20} N_2 O_3$	Prolylleucine	0.14%	0.16%	0.26%
3.578	112.05237	$C_6 H_8 O_2$	Sorbic acid	-	0.24%	-
3.578	390.11607	$C_{16} H_{22} O_{11}$	2,3,4,5,6-Penta-O-acetyl-D-glucose	-	-	0.02%
3.583	342.09523	$C_{15} H_{18} O_9$	Glucocaffeic acid	-	0.01%	-
3.696	276.0269	$C_{14} H_{12} O_2 S_2$	5-(4-Acetoxy-1-butynyl)-2,2'-bithiophene	-	0.01%	-
3.862	162.03152	$C_9 H_6 O_3$	3 Hydroxycoumarin	0.01%	-	-
4.102	366.14233	$C_{17} H_{22} N_2 O_7$	2-{2-[5-(Ethoxycarbonyl)-2-morpholinoanilino]-2-oxoethoxy} acetic acid	0.04%	-	-
4.138	312.04826	$C_{13} H_{12} O_9$	Caftaric acid	T	0.08%	-
4.378	348.08452	$C_{17} H_{16} O_8$	3,5,7,3',4',5'-Hexahydroxy-6,8-dimethylflavanone	0.03%	-	-
4.4	277.05821	$C_{13} H_{11} N O_6$	Salfredin C1	0.14%	-	0.03%
4.78	262.13139	$C_{14} H_{18} N_2 O_3$	5-(tert-butyl)-2-methyl-N-(5-methyl-3-isoxazolyl)-3-furamide	0.06%	-	-
4.847	184.03703	$C_8 H_8 O_5$	Methyl gallate	0.04%	1.05%	-
4.969	176.06842	$C_7 H_{12} O_5$	2-Isopropylmalic acid	-	0.01%	-
4.992	484.08533	$C_{20} H_{20} O_{14}$	1,6-Bis-O-(3,4,5-trihydroxybenzoyl) hexopyranose	6.52%	2.16%	3.78%
5.167	360.10554	$C_{15} H_{20} O_{10}$	Glucosyringic acid	0.08%	0.09%	-
5.539	216.08974	$C_{12} H_{12} N_2 O_2$	L-1,2,3,4-Tetrahydro-beta-carboline-3-carboxylic acid	-	0.02%	-
5.638	444.19969	$C_{21} H_{32} O_{10}$	Cynaroside A	0.02%	0.02%	-
5.779	478.07474	$C_{21} H_{18} O_{13}$	6-Hydroxyluteolin 6-glucuronide	0.04%	0.03%	-

5.919	432.16322	$C_{19}H_{28}O_{11}$	Zizybeoside I	-	0.01%	-
6.16	168.04204	$C_8H_8O_4$	Isovanillic acid	0.08%	0.08%	-
6.538	187.0632	$C_{11}H_9NO_2$	Indoleacrylic acid	-	0.02%	-
6.726	136.05239	$C_8H_8O_2$	4-Methoxybenzaldehyde	0.12%	0.11%	-
6.909	404.13157	$C_{17}H_{24}O_{11}$	Oleoside 11-methyl ester	T	0.03%	-
6.911	230.10533	$C_{13}H_{14}N_2O_2$	(1xi,3xi)-1,2,3,4-Tetrahydro-1-methyl-beta-carboline-3-carboxylic acid	0.04%	-	-
7.074	272.10472	$C_{16}H_{16}O_4$	4'-O-Methyldavidigenin	0.03%	T	-
7.513	196.0733	$C_{10}H_{12}O_4$	Cantharidin	0.10%	0.11%	-
8.848	636.09627	$C_{27}H_{24}O_{18}$	1,3,4-Trigalloyl-β-D-glucopyranose	-	0.07%	0.25%
8.961	478.07436	$C_{21}H_{18}O_{13}$	Quercetin 7-glucuronide	-	-	0.49%
8.973	326.10005	$C_{15}H_{18}O_8$	Melilotoside	0.08%	0.03%	-
8.981	206.05798	$C_{11}H_{10}O_4$	Eugenitol	0.10%	0.03%	0.03%
9.029	338.06378	$C_{15}H_{14}O_9$	Umbelliferone glucuronide	-	0.11%	-
9.206	107.03707	C_6H_5NO	2-Pyridinecarboxaldehyde	-	0.03%	-
9.316	470.01176	$C_{21}H_{10}O_{13}$	Sanguisorbic acid dilactone	0.56%	0.61%	0.19%
9.343	184.09991	$C_{12}H_{12}N_2$	Harmalan	T	-	-
9.501	196.14612	$C_{12}H_{20}O_2$	Dihydro-5-(2-octenyl)-2(3H)-furanone	-	-	0.01%
9.567	634.08082	$C_{27}H_{22}O_{18}$	Sanguiin H4	-	5.63%	0.54%
9.81	308.05281	$C_{14}H_{12}O_8$	Fulvic acid	0.03%	0.06%	-
9.811	478.07452	$C_{21}H_{18}O_{13}$	Miquelianin	0.37%	0.60%	0.39%
9.818	98.03669	$C_5H_6O_2$	2-Furanmethanol	-	0.21%	-
9.876	246.01651	$C_{13}H_{10}OS_2$	Arctinone	-	0.01%	-
9.902	152.04703	$C_8H_8O_3$	Vanillin	0.16%	0.17%	0.17%
9.92	260.03188	$C_{13}H_8O_6$	Urolithin D	-	0.02%	-
9.921	126.03146	$C_6H_6O_3$	Phloroglucinol	0.07%	0.24%	0.27%
10.043	636.09614	$C_{27}H_{24}O_{18}$	1,2,6-Trigalloyl-beta-D-glucopyranose	0.11%	1.42%	2.22%
10.158	634.08037	$C_{27}H_{22}O_{18}$	Corilagin	-	0.15%	-
10.191	242.07893	$C_{11}H_{14}O_6$	Genipinic acid	0.03%	-	-
10.256	296.05303	$C_{13}H_{12}O_8$	Caffeoylmalic acid	0.22%	0.02%	0.06%
10.256	224.03176	$C_{10}H_8O_6$	Dehydrochorismic acid	0.02%	-	-
10.29	233.06862	$C_{12}H_{11}NO_4$	Casimiroin	T	-	-
10.419	96.02088	$C_5H_4O_2$	2H-Pyran-2-one	0.05%	-	-

10.419	484.08522	$C_{20} H_{20} O_{14}$	Hamamelitannin	0.02%	0.11%	-
10.421	126.0316	$C_6 H_6 O_3$	Pyrogallol	6.29%	3.82%	4.87%
10.422	296.05248	$C_{13} H_{12} O_8$	cis-Coutaric acid	0.31%	0.93%	1.05%
10.445	262.01122	$C_{13} H_{10} O_2 S_2$	Arctinone A	-	0.03%	-
10.476	636.09612	$C_{27} H_{24} O_{18}$	1,4,6-Trigalloyl-beta-D-glucopyranose	-	-	0.63%
10.55	601.99658	$C_{28} H_{10} O_{16}$	Diellagilactone	1.13%	0.60%	-
10.68	211.11067	$C_{13} H_{13} N_3$	N,N'-Diphenylguanidine	0.03%	-	-
10.668	448.10005	$C_{21} H_{20} O_{11}$	(1ξ)-1,5-Anhydro-1-[2-(3,4-dihydroxyphenyl)-5,7-dihydroxy-4-oxo-4H-chromen-8-yl]-D-galactitol	-	-	0.06%
10.754	636.09608	$C_{27} H_{24} O_{18}$	1,3,6-Tri-O-galloyl-beta-D-glucose	0.64%	-	-
10.785	198.05265	$C_9 H_{10} O_5$	Syringic acid	0.35%	0.44%	0.48%
10.885	306.03729	$C_{14} H_{10} O_8$	2-(3,4-Dihydroxybenzoyloxy)-4,6-dihydroxybenzoate	-	0.45%	-
10.967	236.03182	$C_{11} H_8 O_6$	6-Methoxy-8-hydroxyisocoumarin-3-carboxylic acid	-	0.07%	0.11%
11.305	170.02138	$C_7 H_6 O_5$	Phloroglucinic acid	0.98%	0.83%	1.24%
11.091	610.1535	$C_{27} H_{30} O_{16}$	Rutin	-	0.03%	0.08%
11.176	432.10539	$C_{21} H_{20} O_{10}$	Vitexin	-	0.05%	-
11.323	162.05261	$C_6 H_{10} O_5$	2-Hydroxyadipic acid	T	0.85%	-
11.361	116.01079	$C_4 H_4 O_4$	Fumaric acid	0.01%	0.63%	-
11.41	194.05781	$C_{10} H_{10} O_4$	Ferulic acid	0.04%	0.02%	-
11.444	236.06825	$C_{12} H_{12} O_5$	1-{4-hydroxy-2-oxo-6-[(1E)-prop-1-en-1-yl]-2H-pyran-3-yl} butane-1,2-dione	0.03%	0.03%	-
11.448	302.04213	$C_{15} H_{10} O_7$	Quercetin	0.02%	-	-
11.485	164.04731	$C_9 H_8 O_3$	2-Hydroxycinnamic acid	-	0.03%	-
11.556	478.11088	$C_{22} H_{22} O_{12}$	6-O-[(2E)-3-(4-Hydroxyphenyl)-2-propenoyl]-1-O-(3,4,5-trihydroxybenzoyl)hexopyranose	-	-	0.14%
11.558	676.23652	$C_{33} H_{40} O_{15}$	Icariin	-	0.01%	-
11.589	170.02147	$C_7 H_6 O_5$	Gallic acid	23.90%	1.90%	1.56%
11.675	264.08989	$C_{16} H_{12} N_2 O_2$	Perlolyrine	-	0.01%	-
11.699	540.22102	$C_{26} H_{36} O_{12}$	Sambacin	-	-	0.01%
11.702	630.12227	$C_{29} H_{26} O_{16}$	Isorhamnetin 3- (6"-galloylglucoside)	-	T	0.01%
11.738	146.05786	$C_6 H_{10} O_4$	2-Methylglutaric acid	-	0.01%	-

11.756	216.0994	$C_{10}H_{16}O_5$	(4S,5S,8S,10R)-4,5,8-trihydroxy-10-methyl-3,4,5,8,9,10-hexahydro-2H-oxecin-2-one	0.06%	-	1.23%
11.822	462.07961	$C_{21}H_{18}O_{12}$	Aureusidin 6-glucuronide	-	-	0.04%
11.905	286.04729	$C_{15}H_{10}O_6$	Maritimetin	T	0.01%	0.01%
11.906	594.15829	$C_{27}H_{30}O_{15}$	Palasitrin	0.01%	-	0.03%
11.921	316.02205	$C_{15}H_8O_8$	2-hydroxyemodic acid	-	0.01%	-
11.938	176.04708	$C_{10}H_8O_3$	4-Methylumbelliferone hydrate	0.03%	0.03%	-
11.949	280.05779	$C_{13}H_{12}O_7$	D-Malic acid p-coumarate	-	-	0.09%
11.953	310.10477	$C_{15}H_{18}O_7$	(E)-1-O-Cinnamoyl-beta-D-glucose	-	-	1.67%
11.955	102.0315	$C_4H_6O_3$	2-Ketobutyric acid	-	-	0.05%
12.024	201.13638	$C_{10}H_{19}NO_3$	Capryloylglycine	-	-	0.04%
12.05	275.11535	$C_{15}H_{17}NO_4$	(±)-Ribaline	0.01%	-	-
12.054	450.16789	$C_{26}H_{26}O_7$	Artonin T	-	T	-
12.095	270.08895	$C_{16}H_{14}O_4$	Isoliquiritigenin 4-methyl ether	T	-	T
12.141	584.1169	$C_{28}H_{24}O_{14}$	2"-O-Galloylisovitexin	-	-	0.01%
12.147	538.20527	$C_{26}H_{34}O_{12}$	Diosbulbinoside F	T	-	-
12.147	256.07309	$C_{15}H_{12}O_4$	Isoliquiritigenin	T	-	-
12.155	436.10094	$C_{20}H_{20}O_{11}$	Taxifolin 3-apioside	-	0.01%	-
12.184	280.09477	$C_{14}H_{16}O_6$	Gravolenic acid	-	T	-
12.185	302.00627	$C_{14}H_6O_8$	Ellagic acid	8.39%	10.46%	11.96%
12.201	220.07336	$C_{12}H_{12}O_4$	Eugenitin	-	-	0.04%
12.233	262.0476	$C_{13}H_{10}O_6$	Maclurin	0.04%	-	-
12.252	126.03158	$C_6H_6O_3$	1,2,3-Trihydroxybenzene	0.10%	0.15%	-
12.254	148.05223	$C_9H_8O_2$	trans-Cinnamic acid	0.76%	0.96%	0.87%
12.255	160.05226	$C_{10}H_8O_2$	6-Methylcoumarin	0.02%	0.01%	-
12.282	374.1364	$C_{20}H_{22}O_7$	8-Hydroxypinoresinol	-	0.26%	-
12.29	318.11033	$C_{17}H_{18}O_6$	Protofarrerol	T	-	0.03%
12.331	174.08908	$C_8H_{14}O_4$	Suberic acid	0.01%	-	0.10%
12.396	262.14158	$C_{12}H_{22}O_6$	Phaseolic acid	0.01%	-	-
12.411	146.03665	$C_9H_6O_2$	Coumarin	0.12%	0.06%	-
12.436	126.10434	$C_8H_{14}O$	(E)-2-octenal	-	-	0.06%
12.48	402.15266	$C_{18}H_{26}O_{10}$	Benzyl beta-primeveroside	0.01%	-	-
12.487	226.06259	$C_{14}H_{10}O_3$	2-Methoxyxanthone	0.01%	-	-

12.492	244.07339	$C_{14} H_{12} O_4$	3,3',4'5-Tetrahydroxystilbene	0.01%	0.01%	-
12.502	114.03157	$C_5 H_6 O_3$	Norfuraneol	0.06%	0.55%	0.08%
12.503	132.0574	$C_9 H_8 O$	Cinnamaldehyde	0.01%	-	0.01%
12.503	192.07847	$C_{11} H_{12} O_3$	(R)-Shinanolone	0.04%	0.03%	0.05%
12.504	310.10483	$C_{15} H_{18} O_7$	(2S,3R,4S,5S,6R)-3,4,5-trihydroxy-6-(hydroxymethyl) oxan-2-yl (2E)-3-phenylprop-2-enoate	2.25%	-	-
12.547	288.06332	$C_{15} H_{12} O_6$	2,4,6-Trihydroxy-2-(4-hydroxybenzyl)-1-benzofuran-3(2H)-one	0.03%	-	-
12.55	344.1256	$C_{19} H_{20} O_6$	Okanin 3,4,3',4'-tetramethyl ether	0.02%	-	-
12.552	374.13624	$C_{20} H_{22} O_7$	3-hydroxy-3,4-bis[(4-hydroxy-3-methoxyphenyl) methyl] oxolan-2-one	0.14%	-	-
12.558	362.17308	$C_{20} H_{26} O_6$	Secoisolariciresinol	-	0.01%	-
12.573	388.11562	$C_{20} H_{20} O_8$	Sigmoidin G	-	T	-
12.593	436.13688	$C_{21} H_{24} O_{10}$	Nothofagin	-	-	0.12%
12.642	150.06799	$C_9 H_{10} O_2$	Hydrocinnamic acid	0.01%	0.03%	0.01%
12.666	646.37157	$C_{36} H_{54} O_{10}$	Gypsogenin 3-O-b-D-glucuronide	-	T	-
12.721	680.37692	$C_{36} H_{56} O_{12}$	Tenuifolin	-	0.11%	0.37%
12.739	550.16884	$C_{26} H_{30} O_{13}$	Licuroside	0.01%	-	-
12.739	442.34453	$C_{29} H_{46} O_3$	4alpha-Methylzymosterol-4-carboxylate	-	T	-
12.745	188.10473	$C_9 H_{16} O_4$	Azelaic acid	0.07%	-	0.35%
12.799	218.05765	$C_{12} H_{10} O_4$	Liqcoumarin	T	0.01%	T
12.8	486.33397	$C_{30} H_{46} O_5$	Bassic acid	-	-	0.05%
12.889	346.1052	$C_{18} H_{18} O_7$	Hamilcone	0.02%	-	0.01%
12.914	190.13544	$C_{13} H_{18} O$	beta-Damascenone	-	0.02%	0.06%
12.915	666.39739	$C_{36} H_{58} O_{11}$	Chebuloside II	-	3.85%	-
12.915	326.15138	$C_{20} H_{22} O_4$	2-[1-(2H-1,3-benzodioxol-5-yl) propan-2-yl]-6-methoxy-4-(prop-2-en-1-yl) phenol	0.03%	0.03%	-
12.929	356.03763	$C_{14} H_{12} O_{11}$	(+)-Chebulic acid	3.98%	1.40%	2.88%
12.967	330.14651	$C_{19} H_{22} O_5$	Hericenone A	-	0.03%	-
12.969	440.32894	$C_{29} H_{44} O_3$	13'-Hydroxy-alpha-tocotrienol	-	-	0.05%
12.969	504.34417	$C_{30} H_{48} O_6$	Madecassic acid	-	-	1.42%
12.971	202.13522	$C_{14} H_{18} O$	(±)-Anisoxide	-	-	0.05%
12.973	244.14583	$C_{16} H_{20} O_2$	Lahorenoic acid C	-	-	0.02%

12.975	674.36638	$C_{37}H_{54}O_{11}$	Cimicifugoside	T	-	-
13.01	358.14147	$C_{20}H_{22}O_6$	Pedicellin	-	0.01%	-
13.081	462.11632	$C_{22}H_{22}O_{11}$	Azalein	-	0.03%	-
13.084	191.09447	$C_{11}H_{13}NO_2$	5-Methoxytryptophol	-	-	0.02%
13.208	288.06318	$C_{15}H_{12}O_6$	Eriodictyol	-	-	0.27%
13.238	148.05233	$C_9H_8O_2$	Cinnamic acid	0.07%	-	0.50%
13.245	360.15695	$C_{20}H_{24}O_6$	Brosimacutin H	0.01%	-	-
13.279	310.12035	$C_{19}H_{18}O_4$	Castillene B	0.01%	-	-
13.354	314.1154	$C_{18}H_{18}O_5$	Crotaoprostrin	-	-	0.01%
13.431	298.08379	$C_{17}H_{14}O_5$	Isoneobavachalcone	T	-	-
13.443	460.13716	$C_{23}H_{24}O_{10}$	7-Hydroxy-5,6-dimethoxyflavone 7-glucoside	-	0.01%	-
13.451	274.08371	$C_{15}H_{14}O_5$	Phloretin	0.01%	0.02%	0.01%
13.536	462.11641	$C_{22}H_{22}O_{11}$	Leptosin	0.01%	0.06%	-
13.562	302.07902	$C_{16}H_{14}O_6$	2,6,3'-Trihydroxy-4'-methoxy-2-benzylcoumaranone	-	0.01%	-
13.59	302.04255	$C_{15}H_{10}O_7$	Bracteatin	0.01%	0.01%	-
13.592	450.07961	$C_{20}H_{18}O_{12}$	Quercetin 4'-galactoside	0.01%	-	-
13.642	514.18403	$C_{27}H_{30}O_{10}$	Baohuoside 1	T	0.01%	-
13.678	568.12178	$C_{28}H_{24}O_{13}$	Isoorientin 2''-p-hydroxybenzoate	-	-	0.04%
13.703	280.13067	$C_{15}H_{20}O_5$	Artabsinolide A	-	-	0.02%
13.805	444.10499	$C_{22}H_{20}O_{10}$	3'-O-Methylderhamnosylmaysin	0.04%	0.12%	0.08%
13.805	292.09447	$C_{15}H_{16}O_6$	(S)-Angelicain	0.05%	-	-
13.806	462.11629	$C_{22}H_{22}O_{11}$	6-O-[(2E)-3-Phenyl-2-propenoyl]-1-O-(3,4,5-trihydroxybenzoyl)-β-D-glucopyranose	0.08%	1.02%	0.18%
13.855	227.09438	$C_{14}H_{13}NO_2$	Koenoline	T	0.02%	-
13.936	614.12731	$C_{29}H_{26}O_{15}$	6-Cinnamoyl-1,2-digalloylglucose	-	0.07%	-
13.971	586.24153	$C_{31}H_{38}O_{11}$	Baccatin III	T	-	0.01%
14.036	664.38246	$C_{36}H_{56}O_{11}$	Medicagenic acid 3-O-beta-D-glucoside	T	-	-
14.066	434.12116	$C_{21}H_{22}O_{10}$	Monospermoside	T	-	-
14.078	534.28285	$C_{29}H_{42}O_9$	Corchoroside A	-	-	0.02%
14.122	578.16359	$C_{27}H_{30}O_{14}$	(1S)-1,5-Anhydro-2-O-(6-deoxy-α-L-mannopyranosyl)-1-[5,7-dihydroxy-2-(4-hydroxyphenyl)-4-oxo-4H-chromen-6-yl]-D-glucitol	0.03%	-	-

14.277	340.13047	$C_{20}H_{20}O_5$	Bakuchalcone	0.01%	-	0.01%
14.358	272.06832	$C_{15}H_{12}O_5$	Naringenin	-	-	0.18%
14.367	470.33907	$C_{30}H_{46}O_4$	Glycyrrhetinic acid	-	0.08%	0.01%
14.413	188.15618	$C_{14}H_{20}$	Congressane		0.01%	0.06%
14.49	614.12725	$C_{29}H_{26}O_{15}$	2-Cinnamoyl-1,6-digalloyl-beta-D-glucopyranose	-	-	0.07%
14.495	148.05227	$C_9H_8O_2$	p-Coumaraldehyde	0.84%	0.15%	0.96%
14.57	614.2364	$C_{32}H_{38}O_{12}$	chrysoeriol 7-O-neohesperidoside	-	T	-
14.618	322.08357	$C_{19}H_{14}O_5$	Ovalitenin C	T	-	-
14.783	234.16167	$C_{15}H_{22}O_2$	Valerenic acid	-	-	0.13%
14.895	518.324	$C_{30}H_{46}O_7$	Perulactone	0.12%	T	0.06%
14.928	202.17175	$C_{15}H_{22}$	Rulepidadiene B	-	-	0.08%
14.93	222.16153	$C_{14}H_{22}O_2$	Rishitin	-	-	0.04%
14.933	330.24009	$C_{18}H_{34}O_5$	9,12,13-TriHOME	-	-	0.05%
15.046	426.09477	$C_{22}H_{18}O_9$	Epiafzelechin 3-O-gallate	-	-	0.01%
15.126	342.11024	$C_{19}H_{18}O_6$	Rengasin trimethyl ether	-	T	-
15.398	470.30287	$C_{29}H_{42}O_5$	17,23-Epoxy-29-hydroxy-27-norlanost-8-ene-3,15,24-trione	T	-	-
15.448	424.33398	$C_{29}H_{44}O_2$	alpha-Tocotrienol	-	0.02%	0.07%
15.454	650.4026	$C_{36}H_{58}O_{10}$	Pedunculoside	-	1.18%	2.61%
15.624	296.10439	$C_{18}H_{16}O_4$	5,7-Dimethoxy-6-C-methylflavone	-	-	0.01%
15.704	316.13099	$C_{18}H_{20}O_5$	O-Methylodoratol	-	T	-
16.257	738.41926	$C_{39}H_{62}O_{13}$	Isonuatigenin 3-[rhamnosyl-(1->2)-glucoside]	-	0.04%	0.12%
16.585	330.24071	$C_{18}H_{34}O_5$	(15Z)-9,12,13-Trihydroxy-15-octadecenoic acid	-	0.03%	0.33%
15.825	346.14158	$C_{20}H_{18}N_4O_2$	Lepidine B	-	-	0.01%
16.852	260.17732	$C_{17}H_{24}O_2$	Falcarindiol	-	-	0.01%
16.854	270.21926	$C_{16}H_{30}O_3$	3-Oxohexadecanoic acid	-	0.01%	-
16.876	252.20856	$C_{16}H_{28}O_2$	Isoambrettolide	-	-	0.32%
16.942	406.28628	$C_{28}H_{38}O_2$	Gymnasterone D	-	0.01%	-
17.155	246.08918	$C_{14}H_{14}O_4$	Marmesin	-	T	-
17.244	288.22977	$C_{16}H_{32}O_4$	(S)-10,16-Dihydroxyhexadecanoic acid	0.03%	-	0.73%
17.275	342.14638	$C_{20}H_{22}O_5$	Brosimacutin C	-	T	-
17.276	180.11471	$C_{11}H_{16}O_2$	Jasmolone	0.05%	-	-
17.30	248.14064	$C_{15}H_{20}O_3$	(S)-Pterosin A	0.02%	-	-

17.31	504.34409	$C_{30}H_{48}O_6$	Protobassic acid	1.26%	0.02%	0.01%
17.33	468.32326	$C_{30}H_{44}O_4$	Glabrolide	0.87%	-	3.19%
17.33	502.32964	$C_{30}H_{46}O_6$	Medicagenic acid	-	T	0.07%
17.331	200.15605	$C_{15}H_{20}$	(S)-gamma-Calacorene	0.02%	-	0.29%
17.363	696.40877	$C_{37}H_{60}O_{12}$	Momordicoside E	-	0.01%	0.04%
17.411	822.40306	$C_{42}H_{62}O_{16}$	Glycyrrhizin	0.01%	-	-
17.489	212.09457	$C_{13}H_{12}N_2O$	Harmine	T	-	0.02%
17.491	284.14093	$C_{18}H_{20}O_3$	(S)-17-Hydroxy-9,11,13,15-octadecatetraynoic acid	-	-	0.13%
17.496	368.16222	$C_{22}H_{24}O_5$	4'-O-Methylxanthohumol	-	T	-
17.595	254.15153	$C_{14}H_{22}O_4$	(2R,5R,6R)-3-[(1E,3E)-hepta-1,3-dien-1-yl]-5,6-dihydroxy-2-(hydroxymethyl) cyclohexan-1-one	-	-	0.46%
17.621	326.11524	$C_{19}H_{18}O_5$	Eucalyptin	-	T	-
17.626	286.08357	$C_{16}H_{14}O_5$	Homobutein	-	-	0.01%
17.635	282.12541	$C_{18}H_{18}O_3$	Ohobanin	T	-	0.06%
17.671	652.27302	$C_{32}H_{44}O_{14}$	Dicrocin	-	-	0.03%
17.847	326.11506	$C_{19}H_{18}O_5$	7-{[(2E)-3-methyl-4-(4-methyl-5-oxo-2,5-dihydrofuran-2-yl) but-2-en-1-yl]oxy}-2H-chromen-2-one	0.02%	-	-
17.946	476.35021	$C_{29}H_{48}O_5$	Homodolichosterone	-	-	T
17.949	224.18904	$C_{13}H_{24}N_2O$	Cuscohygrine	-	0.28%	-
17.956	141.11525	$C_8H_{15}NO$	(R)-Pelletierine	-	-	0.02%
18.237	312.13615	$C_{19}H_{20}O_4$	Desmosdumotin C	T	-	0.02%
18.269	268.14619	$C_{18}H_{20}O_2$	Diethylstilbestrol	-	-	0.02%
18.292	546.35535	$C_{32}H_{50}O_7$	Hovenidulcigenin B	0.04%	-	-
18.339	236.1776	$C_{15}H_{24}O_2$	Capsidiol	-	-	0.02%
18.346	268.13069	$C_{14}H_{20}O_5$	Kamahine C	-	0.01%	0.15%
18.363	336.0994	$C_{20}H_{16}O_5$	Ciliatin A	-	0.01%	-
18.455	328.2249	$C_{18}H_{32}O_5$	Corchorifatty acid F	0.05%	0.01%	0.24%
18.498	340.09447	$C_{19}H_{16}O_6$	Ambanol	-	0.01%	-
18.501	314.2455	$C_{18}H_{34}O_4$	(+/-)9,10-dihydroxy-12Z-octadecenoic acid	-	-	0.30%
18.507	484.31803	$C_{30}H_{44}O_5$	Liquoric acid	T	0.01%	0.04%
18.623	488.35017	$C_{30}H_{48}O_5$	Pitheduloside I	T	0.06%	0.11%
18.792	278.22417	$C_{18}H_{30}O_2$	α-Eleostearic acid	0.02%	0.03%	0.35%

19.621	272.14089	$C_{17}H_{20}O_3$	(S)-Verimol F	0.01%	-	-
19.641	226.09903	$C_{15}H_{14}O_2$	7-Hydroxyflavan	0.03%	-	0.05%
23.2	410.31819	$C_{28}H_{42}O_2$	gamma-Tocotrienol	-	-	0.06%
**Grand Total**				**100%**	**100%**	**100%**
